# Evidence for the additions of clustered interacting nodes during the evolution of protein interaction networks from network motifs

**DOI:** 10.1186/1471-2148-11-133

**Published:** 2011-05-20

**Authors:** Zhongyang Liu, Qijun Liu, Hanchang Sun, Lin Hou, Hao Guo, Yunping Zhu, Dong Li, Fuchu He

**Affiliations:** 1 State Key Laboratory of Proteomics, Beijing Proteome Research Center, Beijing Institute of Radiation Medicine, 27 Taiping Road, Beijing 100850, China; 2 Department of Chemistry and Biology, College of Science, National University of Defense Technology, 109 Deya Road, Changsha 410073, China; 3 Department of Automatic Control, College of Mechatronics and Automation, National University of Defense Technology, 109 Deya Road, Changsha 410073, China; 4 School of Mathematical Sciences, Peking University, 5 Yiheyuan Road, Beijing 100871, China; 5 Institutes of Biomedical Sciences, Fudan University, 130 Dong-An Road, Shanghai 200032, China

## Abstract

**Background:**

High-throughput screens have revealed large-scale protein interaction networks defining most cellular functions. How the proteins were added to the protein interaction network during its growth is a basic and important issue. Network motifs represent the simplest building blocks of cellular machines and are of biological significance.

**Results:**

Here we study the evolution of protein interaction networks from the perspective of network motifs. We find that in current protein interaction networks, proteins of the same age class tend to form motifs and such co-origins of motif constituents are affected by their topologies and biological functions. Further, we find that the proteins within motifs whose constituents are of the same age class tend to be densely interconnected, co-evolve and share the same biological functions, and these motifs tend to be within protein complexes.

**Conclusions:**

Our findings provide novel evidence for the hypothesis of the additions of clustered interacting nodes and point out network motifs, especially the motifs with the dense topology and specific function may play important roles during this process. Our results suggest functional constraints may be the underlying driving force for such additions of clustered interacting nodes.

## Background

In the post-genomic era, the study of networks has obtained unprecedented attention and network-based analyses have played fundamental roles in biological research. Indeed, most genes and proteins function through a complex network between them rather than on their own [1]. Recently, advances in high-throughput experimental technologies have made an ever-increasing amount of data on protein interaction networks (PINs) available. PINs provide a novel perspective for the study of the principles driving the evolution of living organisms.

In the study of the evolution of PINs, one of the most basic and important problems is to explore how the PIN originated and grew. Many researchers have tried to answer the question by multiple approaches. By the theoretical modeling, several evolutionary models of PINs have been established [2-10]. By the analyses on real PINs, several interesting and possible mechanisms have been uncovered [11-16]. Based on the finding that proteins of similar phylogenetic profiles tend to interact with each other, Qin *et al. *for the first time presented the hypothesis that the evolution of PINs has undergone the additions of clustered nodes [12].

Previous studies on the evolution of PINs focus either on the individual protein level [11,17-27], interaction level [11,14,28-30], functional module level [9,15,31-37] or the whole network level [2-8,10,13,16]. Few study the evolution of PINs from the perspective of network motifs [38,39]. Network motifs are referred to as recurring interconnected patterns of specific topology in complex networks, and may represent the simplest building blocks of cellular machines [38,40]. Meanwhile motifs are found to be evolutionarily conserved topological units of cellular networks, which suggests that they are of biological significance [38]. Further, compared with functional modules [41], owing to the definite definition of motifs, they can be explicitly identified and enumerated in various cellular networks [40].

Considering the advantages of network motifs, in this paper, we explore the evolution of PINs from the perspective of network motifs, and try to provide further evidence for the hypothesis that the evolution of PINs has undergone the additions of clustered interacting proteins. First, we classify proteins based on their original time, and analyze the tendency between proteins of the same/different age classes to form motifs in the PIN. Further we investigate whether co-origins of motif constituents are affected by motif topologies and biological functions. Then we focus on those age-homogeneous motifs whose constituents are of the same age class, and analyze the evolution and functions of their members. Finally we discuss how our findings support the hypothesis of the clustered additions and the underlying driving force of the clustered additions.

## Results

### The tendency between proteins of the same/different age classes to form motifs

To understand the evolutionary history of PINs from the network motif perspective, we first analyze the tendency between proteins of the same/different age classes to form motifs in the PIN.

We classify proteins based on their original ages. In our work, we use orthologous groups of orthoMCL [42] to construct the phylogenetic profile and further to assess the original age of the protein. Each orthologous group of orthoMCL is composed of orthologs and only "recent paralogs" whose sequences are similar and thus functions are likely to remain similar. "Ancient paralogs" whose sequences have diverged and thus functions are likely to diverge are assigned into different orthologous groups, and thus their ages are assessed separately. Therefore, using this method, we can crudely assign the original age of a protein to the time when it obtained today's function. Actually, there is no single, optimal method to define the original age of a protein, especially for the protein derived from duplication which is a big source of new gene origins [43,44]. On the one hand, even though we can crudely assess the time when the duplication event happened, in most cases it doesn't make sense to distinguish which copy is the ancestral one and which copy is the created one from this duplication [45]. Therefore, it seems improper to assign the original age of one of the duplicates or both of them to the time when the duplication event happened. On the other hand, for the research on the growth of PINs, it is also improper to assign the original age of all proteins derived from the direct or indirect duplication of a common traceable earliest ancestral protein to the time when the traceable earliest ancestor emerged, because new proteins directly or indirectly from the ancestor are continuously produced at various stages during the evolution of PINs after this ancestor was created. And these today's descended proteins are likely to have been functionally significantly divergent from each other and from the ancestor. Therefore, in our work, we try to define the origin of a protein, taking the phylogeny and meanwhile the (sequence and) function as reference. Especially for a protein from duplication, when it evolved to obtain significantly divergent sequence and function from its ancestor, it is thought to be new. This definition of the original age simply takes sequences and functions as reference, which not only avoids the troublesome reconstruction of the original and evolutionary process of proteins, especially proteins from duplication, but also provides us opportunities to infer the evolutionary process of today's PINs from the functional perspective.

**Figure 1:**
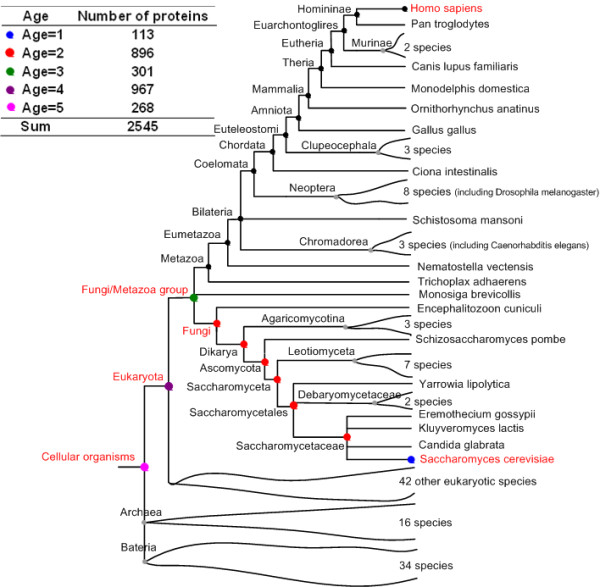
**Schematic representation of the age classification of proteins**. We classify the yeast proteins into 5 age classes based on the phylogenetic relationship of 138 species [46]. Inner nodes on the evolutionary tree represent ancestral organisms and inner nodes on the path from root to *S. cerevisiae *indicate representative time points when the yeast proteins originated during evolution. The path that leads to *S. cerevisiae *is highlighted in bold and 5 age classes are labeled with different colors. The inset table shows the age class distribution of the yeast proteins in the PIN of DIP_YEAST_CORE. The inner nodes on the path from root to *H. sapiens *are also labeled. For the age classification of human proteins, please refer to Supplementary Methods and Results.

As shown in Figure 1, we classify the yeast proteins into 5 age classes based on taxonomy [46]. The most ancient yeast proteins with age 5 are those which originated in the common ancestor of three domains of tree of life (Eukaryota, Bacteria and Archaea) (cellular organisms class: node Cellular organisms). Proteins of the second class with age 4 are those whose traced ancestors appeared before the radiation of eukaryota (and after the radiation of the common ancestor of life) (eukaryota class: node Eukaryota). Those with age 3 emerged before the split of fungi and other fungi/metazoa (fungi/metazoa class: node Fungi/Metazoa group). Those of the fourth class evolved before the split of *S. cerevisiae *and other fungi (fungi class: node Fungi, node Dikarya, node Ascomycota, node Saccharomyceta, node Saccharomycetales and node Saccharomycetaceae). The youngest class contains proteins found only in *S. cerevisiae *(yeast class).

**Figure 2:**
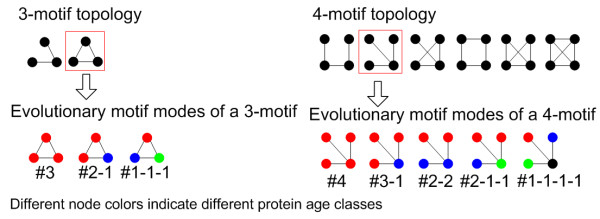
**Network motifs and evolutionary motif modes**. There are two interconnected patterns for 3-motifs and six for 4-motifs. Evolutionary motif modes of a 3-motif and a 4-motif of specific topology are shown, different node colors indicating different protein age classes. For example, for each 4-motif of specific topology, in total there are five possible evolutionary motif modes which are marked as #4, #3-1, #2-2, #2-1-1 and #1-1-1-1. The label for an evolutionary motif mode indicates the number of nodes of different age classes within the motif mode. For example, #4 indicates that all the four proteins within the motif mode are of the same age class, and #2-2 indicates that two of the four proteins within the motif mode are of one age class, while the other two are of another age class.

To study the interconnection tendency between protein nodes of the same/different age classes, based on network motifs, we define "evolutionary motif modes" to characterize particular interconnected patterns of proteins of the same/different age classes (Figure 2). We compute empirical *P *-value for each kind of evolutionary motif mode with specific topology to check the statistical significance of its enrichment or depletion in the real PIN (see Methods). Based on the credible yeast PIN of DIP_YEAST_CORE [47], we find that for the motifs with specific topology, the number of evolutionary motif modes ranges from enrichment to depletion as their constituents gradually change from those of the same age class to those of different age classes (Table 1). The results indicate that in the PIN, proteins of the same age class tend to interact with each other and further to cluster into motifs, while proteins of different age classes tend to avoid interacting with each other and further to avoid forming motifs.

**Table 1:** Interconnection tendency of proteins of the same/different age classes in the PIN of DIP_YEAST_CORE

	Empirical *P- *value ^a ^				
Binary interaction	#2 ^b ^			#1-1	

	**<10^-3 ^**			**(< 10^-3 ^) **	

3-motif	#3		#2-1		#1-1-1

	**<10^-3 ^**		0.455		**(<10^-3 ^) **
	**<10^-3 ^**		1.000		**(<10^-3 ^) **

4-motif	#4	#3-1	#2-2	#2-1-1	#1-1-1-1

	**<10^-3 ^**	**<10^-3 ^**	0.111	**(<10^-3 ^) **	**(<10^-3 ^) **
	**<10^-3 ^**	**<10^-3 ^**	0.758	**(<10^-3 ^) **	**(<10^-3 ^) **
	**<10^-3 ^**	**<10^-3 ^**	0.998	**(<10^-3 ^) **	**(<10^-3 ^) **
	**<10^-3 ^**	**<10^-3 ^**	0.758	**(<10^-3 ^) **	**(<10^-3 ^) **
	**<10^-3 ^**	**<10^-3 ^**	0.994	**(<10^-3 ^) **	**(<10^-3 ^) **
	**<10^-3 ^**	**0.017 **	0.980	**(<10^-3 ^) **	**(<10^-3 ^) **

5-motif ^c ^	#5	#4-1	#2-1-1-1	#1-1-1-1-1	

	**<10^-3 ^**	**<10^-3 ^**	**(<10^-3 ^) **	**(<10^-3 ^) **	
	**<10^-3 ^**	**<10^-3 ^**	**(<10^-3 ^) **	**(<10^-3 ^) **	
	**<10^-3 ^**	**<10^-3 ^**	**(<10^-3 ^) **	**(<10^-3 ^) **	
	**<10^-3 ^**	**<10^-3 ^**	**(<10^-3 ^) **	**(<10^-3 ^) **	
	**<10^-3 ^**	**<10^-3 ^**	**(<10^-3 ^) **	**(<10^-3 ^) **	
	**<10^-3 ^**	0.114	**(<10^-3 ^) **	**(<10^-3 ^) **	

^a ^For #2, #3, #2-1, #4, #3-1, #2-2, #5 and #4-1, upper-tailed *P *-values (enrichment) are listed, and for the other evolutionary motif modes, lower-tailed *P *-values (depletion) in the parentheses are listed. Those lower than 0.05 are highlighted in bold. ^b ^Labels for evolutionary motif modes (Figure 2). ^c ^Considering the length limitation of the table, here for 5-motif we only show four representative motif modes of six representative kinds of topology. Actually for all possible motif modes and topologies, the results are consistent (additional file 1: Table S1).

We obtain the similar results on other PIN datasets, such as YEAST_HC [10], HPRD_HUMAN_HIGH [48], DIP_YEAST [47] and HPRD_HUMAN_ALL [48] (see additional file 1: Table S2, S3, S4, S5, S6, S7, S8 and S9), of which the last two datasets are not well qualitatively controlled and thus are of relatively low quality. The similar results across different datasets indicate that the conclusion above is robust on different data quality and even different organisms.

Here we group ten representative time points into five age classes for yeast based on taxonomy (Figure 1). Actually all the conclusions in this paper keep unchanged across different classifications of age groups (see additional file 1: Supplementary Results and Table S17, S18, S19, S20, S21, S22, S23, S24, S25, S26, S27, S28, S29, S30, S31, S32). In addition, as we know, many ribosomal proteins are evolutionarily conserved and old. The ribosomal proteins in the PIN may influence our results. We find that when removing the ribosomal proteins annotated by FunCat [49] from the PIN of DIP_YEAST_CORE, all the results in the paper still hold (see additional file 1: Table S33, S34, S35, S36, S37, S38, S39 and S40).

### The influence of topologies and biological functions on co-origins of motif constituents

Proteins of the same age class tend to form motifs, while those of different age classes tend to avoid forming motifs. This finding means that in the PIN, age homogeneity of motif constituents is higher than random expectation. In this part we further analyze whether age homogeneity of motif constituents is different for different classes of motifs with special topology or/and function in the real PIN. For this purpose, we introduce the "age homogeneity rate" and the "age homogeneity ratio". The "age homogeneity rate" is referred to as the fraction of motifs whose constituents are of the same age class among a class of motifs with specific topology or/and function. The "age homogeneity ratio" is defined as the ratio of the age homogeneity rate of the real network to its random expectation, which can measure the extent to which a class of motifs with specific topology or/and function affect co-origins of their constituents.

We observe that in the PIN of DIP_YEAST_CORE, motifs with different topologies indeed have different age homogeneity rates (chi-square test, *P *<10^-4 ^for 3, 4, 5-motifs), while this phenomena is absent in random networks (Table 2). Especially, among the motifs with a special number of nodes, the age homogeneity rates seem to be correlated with the topological saturation (Table 2). To quantify this relationship, we test the correlation between motifs' topological saturation (which is simply measured by the number of edges within the motifs) and their age homogeneity (see additional file 1: Table S11), and the correlation between the clustering coefficient and age homogeneity for single proteins (which is defined as the fraction of its interaction partners which are of the same age class as the protein) (see additional file 1: Figure S1). In both cases we observe week but significant positive correlations. Furthermore, by analyzing the age homogeneity ratio, we find that the constraints of motifs with a special number of nodes and edges forcing their constituents' co-origins seem to rise as the number of nodes and edges increases.

**Table 2:** Constraints of topologies on the co-origins of motif constituents

motif ^c ^	The total number	Age homogeneity rate (%) ^a ^		Age homogeneity ratio ^b ^
			
		Real network	Average of 1000 random networks	
	5611	43.11	29.51	1.46
	50536	22.60	10.12	2.23
	2620	39.58	10.05	3.94
	400510	10.03	3.63	2.77
	331797	13.71	3.65	3.76
	4746	16.90	3.65	4.63
	55692	24.53	3.64	6.74
	5748	35.11	3.66	9.58
	1315	39.92	3.61	11.05
	504884	11.37	1.32	8.64
	4237	12.20	1.33	9.19
	399622	13.39	1.33	10.05
	71141	18.79	1.34	14.01
	9125	31.93	1.34	23.90
	632	40.82	1.28	31.87

^a ^Age homogeneity rate is referred to as the fraction of the motifs whose constituents are of the same age class. ^b ^Age homogeneity ratio is defined as the ratio of the age homogeneity rate of the real network to its random expectation which is calculated as the average age homogeneity rate of the 1000 random networks (the fourth column). ^c ^Considering the length limitation of the table, here we only show six representative kinds of topology for 5-motif. Actually for all possible topologies of 5-motifs, the results are consistent (additional file 1: Table S10).

To find out whether the biological functions of the yeast proteins within the motifs affect their age homogeneity, here we only take those motifs whose constituents share at least one common functional category into account, and assign such motifs to the common functional class. First, we find the conclusion that the age homogeneity of motif constituents is higher than random expectation holds for most classes of motifs with specific function (Table 3). Further, we find different biological functions have different age homogeneity rates (chi-square test, *P *<10^-4 ^for 3, 4-motifs) and age homogeneity ratios: motifs belonging to functional classes of protein fate, protein synthesis, and transcription tend to have high age homogeneity ratios, while those belonging to functional classes of energy, signal transduction and metabolism low co-original constraints.

**Table 3:** Constraints of functions on the co-origins of motif constituents

Functional Category ^a ^	The total number	Age homogeneity rate (%) ^c ^		Empirical *P *-value ^b ^	Age homogeneity ratio ^c ^
				
		Real network	Average of 1000 random networks		
3-motif					

Metabolism	3201	14.53	10.15	**0.044 **	1.43
Energy	270	10.00	10.25	0.436	0.98
Cell cycle	5594	21.15	10.11	**<10^-3 ^**	2.09
Transcription	6784	33.89	10.04	**<10^-3 ^**	3.37
Protein synthesis	305	34.43	10.32	**0.001 **	3.34
protein fate	7173	37.82	10.19	**<10^-3 ^**	3.71
Binding protein	4755	26.71	10.14	**<10^-3 ^**	2.63
Regulation of metabolism	96	25.00	10.23	**0.021 **	2.44
Cellular transport	6811	29.14	10.11	**<10^-3 ^**	2.88
Signal transduction	399	14.29	10.32	0.137	1.38
Cell defense	571	18.91	10.15	**0.008 **	1.86
environment interaction	759	16.34	10.16	**0.033 **	1.61
Cell fate	1505	19.73	10.12	**0.003 **	1.95
Cellular components	3521	21.02	10.10	**<10^-3 ^**	2.08
Cell differentiation	2378	18.84	10.10	**0.002 **	1.87

4-motif					

Metabolism	22981	5.07	3.75	0.178	1.35
Energy	949	1.79	3.65	0.641	0.49
Cell cycle	57466	8.57	3.61	**0.019 **	2.37
Transcription	53928	24.05	3.66	**<10^-3 ^**	6.58
Protein synthesis	765	18.95	3.64	**0.002 **	5.21
protein fate	61470	29.36	3.66	**<10^-3 ^**	8.02
Binding protein	29905	17.69	3.64	**<10^-3 ^**	4.86
Regulation of metabolism	142	14.79	3.85	**0.025 **	3.84
Cellular transport	61230	17.93	3.64	**<10^-3 ^**	4.93
Signal transduction	1397	5.01	3.69	0.238	1.36
Cell defense	1889	8.31	3.72	**0.038 **	2.23
environment interaction	2486	6.60	3.80	0.091	1.74
Cell fate	9484	8.31	3.65	**0.016 **	2.28
Cellular components	24647	9.39	3.59	**0.001 **	2.62
Cell differentiation	17140	8.05	3.61	**0.007 **	2.23

^a ^Protein functional categories are based on FunCat functional classification system [49]. Here we list their abbreviations and please refer to Table S12 in additional file 1 for the details. ^b ^We list upper-tailed empirical *P *-values (enrichment), which are lower than 0.05 are highlighted in bold. ^c ^Please refer to the footnotes of Table 2 for the definitions of age homogeneity rate and age homogeneity ratio.

Finally, we also check the joint impact of motif topologies and functions on co-origins of motif constituents (see additional file 1: Table S13). We find the conclusion that age homogeneity of motif constituents is higher than random expectation is also true for most classes of motifs with specific function and topology. Different combinations of biological functions and topologies have different joint constraints forcing co-origins of motif constituents based on their age homogeneity ratios.

### Evolutionary rates and functions of the proteins within motifs whose constituents are of the same age class

To further analyze the evolutionary history of the PIN from network motifs, we focus on those age-homogeneous motifs whose constituents are of the same age class and analyze them from the following aspects.

**Figure 3:**
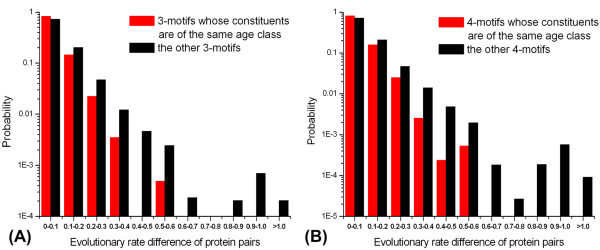
**Distributions of evolutionary rate difference of protein pairs within the age-homogeneous motifs and the other motifs**. The probability (y-axis) is calculated as the percentage of protein pairs whose evolutionary rate difference falls in a special interval that x-axis shows. **(A) **3-motif. Average evolutionary rate difference is 5.8 × 10^-2 ^for 3-motifs whose constituents are of the same age class and 7.9 × 10^-2 ^for the other 3-motifs. Rank sum test, *P *<10^-4 ^. **(B) **4-motif. The average evolutionary rate difference is 6.0 × 10^-2 ^and 8.0 × 10^-2 ^for the two 4-motif classes. Rank sum test, *P *<10^-4 ^. The common protein pairs of the two motif classes are removed in the analyses. The results are based on the PIN of DIP_YEAST_CORE.

First, by computing the evolutionary rates, we find the proteins within the age-homogeneous motifs co-evolve to a significantly higher degree than those participating in the other motifs (Figure 3A, B). Then, we further observe that the constituents of these motifs with constituents of the same age class tend to share the same biological functions (Table 4). From the other point of view, the proteins within the motifs whose members share at least one common functional category tend to be of the same age class, compared with those within the other motifs (see additional file 1: Table S14). Further, compared with the other motifs, these age-homogeneous motifs tend to be within protein complexes (see additional file 1: Table S15). Finally, we find these motifs also tend to have dense intraconnectedness (see additional file 1: Table S16), which is consistent with the finding that the motifs of high topological saturation tend to be of high age homogeneity (Table 2 and Table S11).

**Table 4:** Functional homogeneity rates of the age-homogeneous motifs and the other motifs

	Motifs whose members are of the same age class		The other motifs		*P *-value (Chi-squared test)
		
	Total number	Functional homogeneity rate ^a ^(%)	Total number	Functional homogeneity rate (%)	
Binary interaction	2419	83.5	3192	73.6	<10^-4 ^
3-motif	12457	65.9	40685	51.0	<10^-4 ^
4-motif	102689	46.6	697119	31.7	<10^-4 ^

^a ^The definition of "functional homogeneity rate" is similar to that of "age homogeneity rate". It is calculated as the number of motifs whose members share at least one common functional category divided by the total number of motifs.

In 2003, Wuchty *et al. *found in yeast, proteins that participate in the motifs are more conserved than those that don't [38]. Here we further find that compared with the other motif constituents, proteins participating in age-homogeneous motifs significantly tend to co-evolve, share the same functions and be densely interconnected, and these motifs tend to be within protein complexes.

## Discussion

### Evidence for the hypothesis of the clustered additions from network motifs

In 2003, based on the finding that proteins of similar phylogenetic profiles tend to interact with each other [12], Qin *et al. *first presented the hypothesis that the evolution of PINs has undergone the additions of clustered nodes. Here we find proteins of the same age class not only tend to interact but also tend to form motifs (Table 1), which presents a more direct support for the hypothesis of the clustered additions. Here, "the addition of clustered interacting proteins during the evolution of PINs" means that several proteins along with the interactions between them originated and joined the PIN during a relatively short period of time.

We further explore the possibility of the clustered additions by discussing two alternative scenarios which could lead to the formation of these today's age-homogenous motifs. One scenario is that these proteins formed motifs just during almost the same period of time when these proteins originated, that is, they were clusteredly added during this period of time, and the other is that the interactions between these constituents gradually appeared during a long period of time after these constituents originated, and ultimately formed today's motifs from separated nodes. From the intuitive and parsimonious view, we support the former one. As we know, protein interactions are frequently conserved across multiple organisms [50,51], which is also the theoretical basis for protein interaction prediction using orthologs [52-56]. In our study, proteins within these age-homogeneous motifs significantly tend to share similar phylogenetic profiles (see additional file 1: Figure S2), which means these proteins significantly co-occur in different genomes. We have already known they form motifs in yeast. Then based on the conservation of interactions, we can speculate that their co-occurring orthologous hits are likely to form motifs in other species. When a motif exists in multiple species, from the most parsimonious perspective, the motif existed in the ancestral species rather than gradually formed in child species independently. This suggests that the proteins within today's age-homogenous motifs formed motifs during almost the same period of time when these proteins originated, that is, they are much more likely to be clusteredly added to the PIN during evolution.

Meanwhile, co-evolution (Figure 3A, B) and functional homogeneity (Table 4 Table S14 and Table S15 in the additional file 1) of the constituents within these age-homogenous motifs are consistent with their clustered additions. It is likely that after these proteins' traced ancestors were clusteredly added to the PIN (maybe as a result of functional needs), they together played a functionally important role, and thus underwent similar inner and outer pressure and co-evolved to further maintain steady motif structure to "guarantee" biological functions.

Our results from network motifs suggest that the proteins within age-homogeneous motifs tend to be clusteredly added historically during a (short) period of time. However such tendencies of clustered additions are affected by topologies and biological functions. Motifs with specific function and dense topology were more likely to be clusteredly added to the PIN (Table 2 and 3).

### The impact of "recent paralogs" on the issue of the clustered additions

In our work, the recent paralogs in an orthologous group which are likely to retain the similar functions will be traced to the same origin and thus be assigned the same original age, which will result in some age-homogeneous motifs in which some members are ("recent") paralogous to other members. The members of such age-homogeneous motifs may not be thought to be clusteredly added to the network during the (short) period of time when these members originated. Because at the original time of these members, there is only one ancestor of these paralogous members and such age-homogeneous motifs' ultimate formation depends on the later (recent) duplication event. However actually we find the fractions of such motifs with recent paralog pairs among all the age-homogeneous motifs are small, which are only 2.4% for 3-motifs and 2.7% for 4-motifs.

### Evidence for the hypothesis of the clustered additions from protein complexes

**Figure 4:**
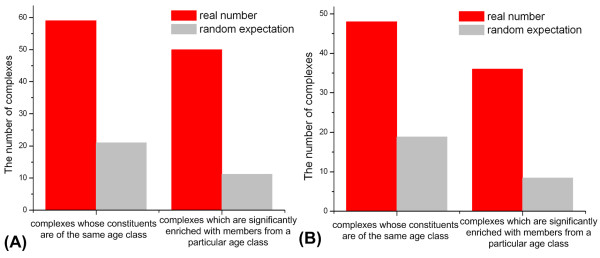
**The number of yeast protein complexes and their random expectation**. We consider two kinds of protein complexes. One is those whose members are all of the same age class, and the other is those which are significantly enriched with members from a particular age class. The random expectation is the average of 1000 randomizations which is established by randomizing the corresponding relationships between proteins in the yeast genome and their ages. The empirical *P *-values are all less than 10^-3 ^. **(A) **The results are obtained considering all yeast protein complexes. **(B) **The results are obtained only considering yeast protein complexes without recent paralog pairs (see the Discussion part for the details).

Another evidence for the additions of clustered interacting nodes comes from the analyses of yeast protein complexes [57]. We find there are significantly more age-homogeneous complexes whose constituents are all of the same age class than random expectation based on 1000 experiments established by randomizing the corresponding relationships between proteins in the yeast genome and their ages. Further, among the other age-heterogeneous complexes, there are also significantly more complexes which are significantly enriched with members from a special age class (the corresponding upper-tailed *P- *value of hypergeometric cumulative distribution [58] is less than 0.05) than random expectation (Figure 4A). These results still hold when only considering protein complexes without recent paralog pairs (see the second part of Discussion for the details) (Figure 4B).

### Functional constraints as the possible driving force of the clustered additions

Qin *et al. *used natural selection to explain the additions of clustered nodes [12]. They thought that a new function likely requires a group of interacting new proteins and the growth of PINs is under functional constraints. Indeed, we find co-evolution (Figure 3A, B) of the constituents of these age-homogeneous motifs, which suggests functional significance for a cluster of interacting proteins. Also we find proteins within these age-homogeneous motifs tend to share the same biological functions (Table 4) and these motifs tend to be within known protein complexes (see additional file 1: Table S15). All the results indicate that these motifs of the same age class tend to be functionally significant. What is more, as we know, protein complexes are definite functional modules in the PIN. Their analytic results (Figure 4) provide powerful evidence for functional constraints as the driving force of the additions of clustered interacting nodes.

## Conclusions

In the PIN, proteins of the same age class tend to form motifs while those of different age classes tend to avoid forming motifs. The constituents within the motifs with specific function or dense topology tend to be under high co-original constraints. Further the proteins participating in the motifs with members of the same age class tend to be densely interconnected, share the same functions and evolve at similar rates, and these motifs tend to be within protein complexes. These results suggest that the age-homogeneous motifs historically tend to be clusteredly added to the PIN, especially those with dense topology and specific function, providing evidence for the hypothesis of the additions of clustered interacting nodes from the network motif perspective for the first time. Our results also suggest functional constraints may be the underlying driving force for such clustered additions.

## Methods

### Protein-protein interactions

For yeast, we use two protein-protein interaction datasets. One is from Database of Interacting Proteins (DIP) which catalogs experimentally determined protein interactions from a variety of sources (Version 20080114) [47]. After removing self-interactions, we obtain 15410 yeast protein interactions between 4551 proteins (DIP_YEAST). Especially, DIP provides a reliable, core subset of DIP_YEAST which is denoted as DIP_YEAST_CORE (Version 20071007). This core subset contains protein interactions that have been computationally verified or observed in more than one large-scale experiment or those that come from small-scale experiments [26]. After self-interactions are removed, DIP_YEAST_CORE contains 5611 interactions between 2545 proteins. To validate the universality of our analytic results, we use the other yeast protein interaction dataset which contains 12051 non-self interactions between 3264 proteins. This dataset denoted as YEAST_HC is from Kim and Marcotte [10] and is a reliable subset of literature-curated yeast protein interaction data in BioGrid [59].

In addition, for testing the robustness of the result of the interconnection tendency between the proteins of the same/different age classes on PINs of other organisms, we also analyze the other two human PINs respectively denoted as HPRD_HUMAN_ALL (high-throughput and low-throughput experimental interactions, 22545 non-self interactions, 6919 proteins) and HPRD_HUMAN_HIGH (low-throughput experimental interactions, 17156 non-self interactions, 5704 proteins), which are downloaded from Human Protein Reference Database (HPRD) (Release 7) [48].

### Yeast protein complexes

We use re-annotated, manually curated MIPS yeast protein complexes provided by de Lichtenberg *et al. *which contain 199 complexes, 966 proteins [57]. Compared with original MIPS complexes [60], the re-annotated data reflect known dynamic expression information of proteins and thus can better represent real complexes *in vivo *. For example, *in vivo *Cdc28p can only interact with a single cyclin at a time, however in MIPS Cdc28p and all its 9 interacting cyclins are organized as a single complex. To correct this, de Lichtenberg *et al. *annotated 9 complexes instead.

### Age assessment of proteins

We use the GeneTrace algorithm with default parameters to assess each protein's original age [61]. GeneTrace is an efficient algorithm that allows the reconstruction of the most likely evolutionary scenario of an individual protein, including the original time of this protein, given a phylogenetic profile of the protein and an evolutionary tree including all organisms involved. Compared with the simple method of finding orthologs in representative species [62-64], GeneTrace algorithm takes gene loss and horizontal transfer events into account to a certain extent, and thus is more precise in assessing protein ages. The phylogenetic profile of a protein is defined as a binary vector based on the presence (1) or absence (0) of its orthologous hits in the reference genomes. Here we use orthologous groups from orthoMCL (Version 4.0) [42] to construct the phylogenetic profiles. Each orthologous group from orthoMCL consists of orthologs and only "recent paralogs" derived from recent gene duplication which retain similar sequences and are likely to retain similar functions. Those "ancient paralogs" from ancient duplication events which are likely to exhibit divergent functions are assigned into different orthologous groups of orthoMCL [42]. Totally, the orthologous group data of orthoMCL involve 50 prokaryotic and 88 eukaryotic genomes and thus the phylogenetic profile here is a 138-dimention binary vector. Phylogenetic tree including these 138 species is from NCBI Taxonomy common tree system (Version 2010 Aug) [46] (Figure 1).

### Network motifs and evolutionary motif modes

"Network motifs" are recurring, topologically distinct interconnected patterns of nodes in complex networks [38,40]. Based on network motifs, we define "evolutionary motif modes" as network motifs which characterize particular interconnected patterns of proteins of the same/different age classes (Figure 2). We use FANMOD software [65] to detect network motifs and then Perl programs to obtain evolutionary motif modes. FANMOD software implements RAND-ESU algorithm to enumerate and sample the vertex-induced motifs [66]. For a given subset of the vertices of network G, the vertex-induced motif is unique. Therefore, there are not motifs with the same vertices but with different topologies. This algorithm is orders of magnitude faster than any other existing algorithms for this task [67].

### Random age assignment and empirical *P *-value

If the ages of proteins don't impact the interconnected patterns of proteins of the same/different age classes in the PIN, a random age assignment should give similar interconnected patterns as seen in the real PIN. To analyze the interconnection tendency of proteins of the same/different age classes, we first generate 1000 random networks by randomizing the corresponding relationships between proteins and their ages in real network. Then we use empirical *P *-value to evaluate the statistical significance of enrichment/depletion of each kind of evolutionary motif mode in the real network [68,69]. For each kind of motif mode of specific topology, the empirical *P *-value is calculated as the fraction of random networks in which its number is not smaller than (upper tail) or not larger than (lower tail) that in real network. The evolutionary motif modes are significantly enriched/depleted in the real network when the upper-tailed/lower-tailed *P *-value is less than 0.05.

### Functional annotation of yeast proteins

The molecular functions of yeast proteins are based on Functional Catalogue (FunCat) annotations [49] from MIPS/CYGD database [60]. FunCat is a hierarchically structured functional classification system, and each FunCat term can be traced to different annotation levels in the hierarchies. Here we only focus on the first level (see additional file 1: Table S12).

### Yeast protein evolutionary rates

The evolutionary rate of a protein is defined as the ratio between the number of non-synonymous substitutions per non-synonymous site (*dN *) and the number of synonymous substitutions per synonymous site (*dS *). To compute evolutionary rates of *S. cerevisiae *proteins, we adopt *S. paradoxus *as reference species which is the most closely related species to *S. cerevisiae *among all the completely sequenced organisms. Amino acid sequences and corresponding coding sequences (CDS) of proteins of the two species are from Saccharomyces Genome Database (SGD) (for *S. cerevisiae *, Version 20-Feb-2009 and for *S. paradoxus *, Version 14-Dec-2004) [70]. *S. cerevisiae-S. paradoxus *orthologs are obtained using Inparanoid program [71]. Pairs of orthologous proteins are aligned using the ClustalW program [72] and *dN */*dS *s are calculated using PAML program [73].

## Abbreviations

CDS: coding sequences; CYGD: Comprehensive Yeast Genome Database; DIP: Database of Interacting Proteins; FunCat, Functional Catalogue; HPRD: Human Protein Reference Database; MIPS: Munich Information Center for Protein Sequences; PIN: protein interaction network; SGD: Saccharomyces Genome Database

## Authors' contributions

ZL designed the study, carried out the study and wrote the manuscript. DL provided guidance and helped write and revise the manuscript. QL, HS, LH and HG participated in the analyses. FH and YZ provided guidance and revised the manuscript. All authors read and approved the manuscript.

## Supplementary Material

Additional file 1 **Supplementary results, methods, tables and figures**. supplementary results, methods, tables (Table S1, S2, S3, S4, S5, S6, S7, S8, S9, S10, S11, S12, S13, S14, S15, S16, S17, S18, S19, S20, S21, S22, S23, S24, S25, S26, S27, S28, S29, S30, S31, S32, S33, S34, S35, S36, S37, S38, S39 and S40) and figures (Figure S1 and S2) Click here for file

## References

[B1] Vespignani A Evolution thinks modular Nat Genet 2003 35 118 119 10.1038/ng1003-118 14517536

[B2] Kim J Krapivsky PL Kahng B Redner S Infinite-order percolation and giant fluctuations in a protein interaction network Phys Rev E Stat Nonlin Soft Matter Phys 2002 66 5 Pt 2 055101 12513542 10.1103/PhysRevE.66.055101

[B3] Chung F Lu L Dewey TG Galas DJ Duplication models for biological networks J Comput Biol 2003 10 677 687 10.1089/106652703322539024 14633392

[B4] Pastor-Satorras R Smith E Sole RV Evolving protein interaction networks through gene duplication J Theor Biol 2003 222 199 210 10.1016/S0022-5193(03)00028-6 12727455

[B5] Vázquez A Flammini A Maritan A Vespignani A Modeling of protein interaction networks Complexus 2003 1 38 44 10.1159/000067642

[B6] Berg J Lässig M Wagner A Structure and evolution of protein interaction networks: a statistical model for link dynamics and gene duplications BMC Evol Biol 2004 4 51 10.1186/1471-2148-4-51 15566577 PMC544576

[B7] Hallinan J Gene duplication and hierarchical modularity in intracellular interaction networks BioSystems 2004 74 51 62 10.1016/j.biosystems.2004.02.004 15125992

[B8] Hormozdiari F Berenbrink P Przulj N Sahinalp SC Not all scale-free networks are born equal: the role of the seed graph in PPI network evolution PLoS Comput Biol 2007 3 e118 10.1371/journal.pcbi.0030118 17616981 PMC1913096

[B9] Pereira-Leal JB Levy ED Kamp C Teichmann SA Evolution of protein complexes by duplication of homomeric interactions Genome Biol 2007 8 R51 10.1186/gb-2007-8-4-r51 17411433 PMC1895999

[B10] Kim WK Marcotte EM Age-dependent evolution of the yeast protein interaction network suggests a limited role of gene duplication and divergence PLoS Comput Biol 2008 4 e1000232 10.1371/journal.pcbi.1000232 19043579 PMC2583957

[B11] Fraser HB Hirsh AE Steinmetz LM Scharfe C Feldman MW Evolutionary rate in the protein interaction network Science 2002 296 750 752 10.1126/science.1068696 11976460

[B12] Qin H Lu HH Wu WB Li WH Evolution of the yeast protein interaction network Proc Natl Acad Sci USA 2003 100 12820 12824 10.1073/pnas.2235584100 14557537 PMC240702

[B13] Wagner A How the global structure of protein interactrion networks evolves Proc R Soc Lond B 2003 270 457 466 10.1098/rspb.2002.2269 PMC1691265 12641899

[B14] Mintseris J Weng Z Structure, function, and evolution of transient and obligate protein-protein interactions Proc Natl Acad Sci USA 2005 102 10930 10935 10.1073/pnas.0502667102 16043700 PMC1182425

[B15] Pereira-Leal JB Teichmann SA Novel specificities emerge by stepwise duplication of functional modules Genome Res 2005 15 552 559 10.1101/gr.3102105 15805495 PMC1074369

[B16] Fernández A Molecular basis for evolving modularity in the yeast protein interaction network PLoS Comput Biol 2007 3 e226 10.1371/journal.pcbi.0030226 17997598 PMC2065896

[B17] Bloom JD Adami C Apparent dependence of protein evolutionary rate on number of interactions is linked to biases in protein-protein interactions data sets BMC Evol Biol 2003 3 21 10.1186/1471-2148-3-21 14525624 PMC270031

[B18] Fraser HB Wall DP Hirsh AE A simple dependence between protein evolution rate and the number of protein-protein interactions BMC Evol Biol 2003 3 11 10.1186/1471-2148-3-11 12769820 PMC166126

[B19] Jordan IK Wolf YI Koonin EV No simple dependence between protein evolution rate and the number of protein-protein interactions: only the most prolific interactors tend to evolve slowly BMC Evol Biol 2003 3 1 10.1186/1471-2148-3-1 12515583 PMC140311

[B20] Bloom JD Adami C Evolutionary rate depends on number of protein-protein interactions independently of gene expression level: Response BMC Evol Biol 2004 4 14 10.1186/1471-2148-4-14 15171796 PMC443507

[B21] Fraser HB Hirsh A Evolutionary rate depends on number of protein-protein interactions independently of gene expression level BMC Evol Biol 2004 4 13 10.1186/1471-2148-4-13 15165289 PMC420460

[B22] Wuchty S Evolution and topology in the yeast protein interaction network Genome Res 2004 14 1310 1314 10.1101/gr.2300204 15231746 PMC442146

[B23] Agrafioti I Swire J Abbott J Huntley D Butcher S Stumpf MP Comparative analysis of the *Saccharomyces cerevisiae *and *Caenorhabditis elegans *protein interaction networks BMC Evol Biol 2005 5 23 10.1186/1471-2148-5-23 15777474 PMC1079807

[B24] Hahn MW Kern AD Comparative genomics of centrality and essentiality in three eukaryotic protein-interaction networks Mol Biol Evol 2005 22 803 806 10.1093/molbev/msi072 15616139

[B25] Drummond DA Raval A Wike CO A single determinant dominates the rate of yeast protein evolution Mol Biol Evol 2006 23 327 337 16237209 10.1093/molbev/msj038

[B26] Saeed R Deane CM Protein protein interactions, evolutionary rate, abundance and age BMC Bioinformatics 2006 7 128 10.1186/1471-2105-7-128 16533385 PMC1431566

[B27] Kim PM Korbel JO Gerstein MB Positive selection at the protein network periphery: Evaluation in terms of structural constraints and cellular context Proc Natl Acad Sci USA 2007 104 20274 20279 10.1073/pnas.0710183104 18077332 PMC2154421

[B28] Teichmann SA The constraints protein-protein interactions place on sequence divergence J Mol Biol 2002 324 399 407 10.1016/s0022-2836(02)01144-0 12445777

[B29] Fraser HB Hirsh AE Wall DP Eisen MB Coevolution of gene expression among interacting proteins Proc Natl Acad Sci USA 2004 101 9033 9038 10.1073/pnas.0402591101 15175431 PMC439012

[B30] Fraser HB Hirsh AE Wall DP Eisen MB Correlation between transcriptome and interactome mapping data from *Saccharomyces cerevisiae *Nat Genet 2004 29 482 426 10.1038/ng776 11694880

[B31] Snel B Huynen MA Quantifying modularity in the evolution of biomolecular systems Genome Res 2004 14 391 397 10.1101/gr.1969504 14993205 PMC353226

[B32] Fraser HB Modularity and evolutionary constraint on proteins Nat genet 2005 37 351 352 10.1038/ng1530 15750592

[B33] Vergassola M Vespignani A Dujon B Cooperative evolution in protein complexes of yeast from comparative analysis of its interaction network Proteomics 2005 5 3116 3119 10.1002/pmic.200401138 16035114

[B34] Batada NN Reguly T Breitkreutz A Boucher L Breitkreutz BJ Hurst LD Tyers M Stratus not altocumulus: a new view of the yeast protein interaction network PLOS Biol 2006 4 e317 10.1371/journal.pbio.0040317 16984220 PMC1569888

[B35] Chen Y Dokholyan NV The coordinated evolution of yeast proteins is constrained by functional modularity Trends Genet 2006 22 416 419 10.1016/j.tig.2006.06.008 16797778

[B36] Batada NN Reguly T Breitkreutz A Boucher L Breitkreutz BJ Hurst LD Tyers M Still stratus not altocumulus: further evidence against the date/party hub distinction PLoS Biol 2007 5 e154 10.1371/journal.pbio.0050154 17564494 PMC1892831

[B37] Bertin N Simonis N Dupuy D Cusick ME Han JD Fraser HB Roth FP Vidal M Confirmation of organized modularity in the yeast interactome PLOS Biol 2007 5 e153 10.1371/journal.pbio.0050153 17564493 PMC1892830

[B38] Wuchty S Oltvai ZN Barabási AL Evolutionary conservation of motif constituents in the yeast protein interaction network Nat Genet 2003 35 176 179 10.1038/ng1242 12973352

[B39] Lee WP Jeng BC Pai TW Tsai CP Yu CY Tzou WS Differential evolutionary conservation of motif modes in the yeast protein interaction network BMC Genomics 2006 7 89 10.1186/1471-2164-7-89 16638125 PMC1501022

[B40] Milo R Shen-Orr S Itzkovitz S Kashtan N Chklovskii D Alon U Network motifs: simple building blocks of complex networks Science 2002 298 824 827 10.1126/science.298.5594.824 12399590

[B41] Hartwell LH Hopfield JJ Leibler S Murray AW From molecular to modular cell biology Nature 1999 402 6761 Suppl C47 52 10591225 10.1038/35011540

[B42] Li L Stoeckert CJ Jr Roos DS OrthoMCL: identification of ortholog groups for eukaryotic genomes Genome Res 2003 13 2178 2189 10.1101/gr.1224503 12952885 PMC403725

[B43] Wolf YI Novichkov PS Karev GP Koonin EV Lipman DJ The universal distribution of evolutionary rates of genes and distinct characteristics of eukaryotic genes of different apparent ages Proc Natl Acad Sci USA 2009 106 7273 7280 10.1073/pnas.0901808106 19351897 PMC2666616

[B44] Domazet-Loso T Tautz D An ancient evolutionary origin of genes associated with human genetic diseases Mol Biol Evol 2008 5 2699 2707 10.1093/molbev/msn214 PMC2582983 18820252

[B45] Han M Hahn M Identifying parent-daughter relationships among duplicated genes Pacific Symposium on Biocomputing 2009 14 114 125 19213133

[B46] Wheeler DL Barrett T Benson DA Bryant SH Canese K Chetvernin V Church DM DiCuccio M Edgar R Federhen S Geer LY Helmberg W Kapustin Y Kenton DL Khovayko O Lipman DJ Madden TL Maglott DR Ostell J Pruitt KD Schuler GD Schriml LM Sequeira E Sherry ST Sirotkin K Souvorov A Starchenko G Suzek TO Tatusov R Tatusova TA Database resources of the National Center for Biotechnology Information Nucleic Acids Res 2006 35 Database D5 12 10.1093/nar/gkl1031 PMC1781113 17170002

[B47] Salwinski L Miller CS Smith AJ Pettit FK Bowie JU Eisenberg D The Database of Interacting Proteins: 2004 update Nucleic Acids Res 2004 32 Database D449 451 10.1093/nar/gkh086 PMC308820 14681454

[B48] Keshava-Prasad TS Goel R Kandasamy K Kandasamy K Keerthikumar S Kumar S Mathivanan S Telikicherla D Raju R Shafreen B Venugopal A Balakrishnan L Marimuthu A Banerjee S Somanathan DS Sebastian A Rani S Ray S Harrys Kishore CJ Kanth S Ahmed M Kashyap MK Mohmood R Ramachandra YL Krishna V Rahiman BA Mohan S Ranganathan P Ramabadran S Chaerkady R Human Protein Reference Database - 2009 update Nucleic Acids Res 2009 37 Database D767 772 10.1093/nar/gkn892 PMC2686490 18988627

[B49] Ruepp A Zollner A Maier D Albermann K Hani J Mokrejs M Tetko I Güldener U Mannhaupt G Münsterkötter M Mewes HW The FunCat, a functional annotation scheme for systematic classification of proteins from whole genomes Nucleic Acids Res 2004 32 5539 5545 10.1093/nar/gkh894 15486203 PMC524302

[B50] Kelley BP Sharan R Karp RM Sittler T Root DE Stockwell BR Ideker T Conserved pathways within bacteria and yeast as revealed by global protein network alignment Proc Natl Acad Sci USA 2003 100 11394 11399 10.1073/pnas.1534710100 14504397 PMC208768

[B51] Pagel P Mewes HW Frishman D Conservation of protein-protein interactions--lessons from ascomycota Trends Genet 2004 20 72 76 10.1016/j.tig.2003.12.007 14746987

[B52] Persico M Ceol A Gavrila C Hoffmann R Florio A Cesareni G HomoMINT: an inferred human network based on orthology mapping of protein interactions discovered in model organisms BMC Bioinformatics 2005 6 Suppl 4 S21 10.1186/1471-2105-6-S4-S21 16351748 PMC1866386

[B53] Rhodes DR Tomlins SA Varambally S Mahavisno V Barrette T Kaly-ana-Sundaram S Ghosh D Pandey A Chinnaiyan AM Probabilistic model of the human protein-protein interaction network Nat Biotechnol 2005 23 951 959 10.1038/nbt1103 16082366

[B54] Huang TW Lin CY Kao CY Reconstruction of human protein interolog network using evolutionary conserved network BMC Bioinformatics 2007 8 152 10.1186/1471-2105-8-152 17493278 PMC1885812

[B55] Brown KR Jurisica I Online predicted human interaction database Bioinformatics 2005 21 2076 2082 10.1093/bioinformatics/bti273 15657099

[B56] Han K Park B Kim H Hong J Park J HPID: the Human Protein Interaction Database Bioinformatics 2004 20 2466 2470 10.1093/bioinformatics/bth253 15117749

[B57] de Lichtenberg U Jensen LJ Brunak S Bork P Dynamic complex formation during the yeast cellular cycle Science 2005 307 724 727 10.1126/science.1105103 15692050

[B58] Zhao J Ding GH Tao L Yu H Yu ZH Luo JH Cao ZW Li YX Modular co-evolution of metabolic networks BMC Bioinformatics 2007 8 311 10.1186/1471-2105-8-311 17723146 PMC2001200

[B59] Stark C Breitkreutz BJ Reguly T Boucher L Breitkreutz A Tyers M BioGRID: a general repository for interaction datasets Nucleic Acids Res 2006 34 Database D535 539 10.1093/nar/gkj109 PMC1347471 16381927

[B60] Mewes HW Frishman D Guldener U Mannhaupt G Mayer K Mokrejs M Morgenstern B Munsterkotter M Rudd S Weil B MIPS: A database for genomes and protein sequences Nucleic Acids Res 2002 30 31 34 10.1093/nar/30.1.31 11752246 PMC99165

[B61] Kunni V Ouzounis CA GeneTRACE-reconstruction of gene content of ancestral species Bioinformatics 2003 19 1412 1416 10.1093/bioinformatics/btg174 12874054

[B62] Li S Armstrong CM Bertin N Ge H Milstein S Boxem M Vidalain PO Han JD Chesneau A Hao T Goldberg DS Li N Martinez M Rual JF Lamesch P Xu L Tewari M Wong SL Zhang LV Berriz GF Jacotot L Vaglio P Reboul J Hirozane-Kishikawa T Li Q Gabel HW Elewa A Baumgartner B Rose DJ Yu H A Map of the Interaction Network of the Metazoan *C.elegans *Science 2004 303 540 543 10.1126/science.1091403 14704431 PMC1698949

[B63] Albà MM Castresana J Inverse relationship between evolutionary rate and age of mammalian genes Mol Biol Evol 2005 22 598 606 15537804 10.1093/molbev/msi045

[B64] Rual JF Venkatesan K Hao T Hirozane-Kishikawa T Dricot A Li N Berriz GF Gibbons FD Dreze M Ayivi-Guedehoussou N Klitgord N Simon C Boxem M Milstein S Rosenberg J Goldberg DS Zhang LV Wong SL Franklin G Li S Albala JS Lim J Fraughton C Llamosas E Cevik S Bex C Lamesch P Sikorski RS Vandenhaute J Zoghbi HY Towards a proteome-scale map of the human protein-protein interaction network Nature 2005 437 1173 1178 10.1038/nature04209 16189514

[B65] Sebastian W Florian R FANMOD: a tool for fast network motif detection Bioinform atics 2006 22 1152 1153 10.1093/bioinformatics/btl038 16455747

[B66] Alon N Dao P Hajirasouliha I Hormozdiari F Sahinalp SC Biomolecular network motif counting and discovery by color coding Bioinformatics 2008 24 i241 249 18586721 10.1093/bioinformatics/btn163 PMC2718641

[B67] Wernicke S R Casadia and G Myers A faster algorithm for detecting network motifs Lecture Notes in Bioinformatics 2005 3692 Heidelberg: Springer Berlin 165 177

[B68] Giot L Bader JS Brouwer C Chaudhuri A Kuang B Li Y Hao YL Ooi CE Godwin B Vitols E Vijayadamodar G Pochart P Machineni H Welsh M Kong Y Zerhusen B Malcolm R Varrone Z Collis A Minto M Burgess S McDaniel L Stimpson E Spriggs F Williams J Neurath K Ioime N Agee M Voss E Furtak K A protein interaction map of Drosophila melanogaster Science 2003 302 1727 1736 10.1126/science.1090289 14605208

[B69] Welch WJ Construction of permutation tests, *Journal of American Statistical Association *1990 85 693 698

[B70] Hirschman JE Balakrishnan R Christie KR Costanzo MC Dwight SS Engel SR Fisk DG Hong EL Livstone MS Nash R Park J Oughtred R Skrzypek M Starr B Theesfeld CL Williams J Andrada R Binkley G Dong Q Lane C Miyasato S Sethuraman A Schroeder M Thanawala MK Weng S Dolinski K Botstein D Cherry JM Genome Snapshot: a new resource at the Saccharomyces Genome Database (SGD) presenting an overview of the Saccharomyces cerevisiae genome Nucleic Acids Res 2006 34 Database D442 445 10.1093/nar/gkj117 PMC1347479 16381907

[B71] O'Brien KP Remm M Sonnhammer EL Inparanoid: a comprehensive database of eukaryotic orthologs Nucleic Acids Res 2005 33 Database D476 480 10.1093/nar/gki107 PMC540061 15608241

[B72] Higgins DG Thompson JD Gibson TJ Using CLUSTAL for multiple sequence alignments Methods Enzymol 1996 266 383 402 8743695 10.1016/s0076-6879(96)66024-8

[B73] Yang Z PAML 4: phylogenetic analysis by maximum likelihood Mol Biol Evol 2007 24 1586 1591 10.1093/molbev/msm088 17483113

