# Supplementary material for: Evidence for the additions of clustered interacting nodes during the evolution of protein interaction networks from network motifs
Source: BMC Evol Biol. 2011 May 20;11:133. doi: 10.1186/1471-2148-11-133 (PMC3128043; doi:10.1186/1471-2148-11-133)
Supplement: Additional file 1 — Supplementary results, methods, tables and figures. supplementary results, methods, tables (Table S1, S2, S3, S4, S5, S6, S7, S8, S9, S10, S11, S12, S13, S14, S15, S16, S17, S18, S19, S20, S21, S22, S23, S24, S25, S26, S27, S28, S29, S30, S31, S32, S33, S34, S35, S36, S37, S38, S39 and S40) and figures (Figure S1 and S2) [file 1471-2148-11-133-S1.PDF]

Supplementary information for

Evidence for the additions of clustered interacting nodes during the evolution of protein interaction networks from network motifs

Table of contents

Supplementary Results

The impact of different age classifications---2

Supplementary Methods

Age classification of human proteins---3

Supplementary Tables and Figures

Table S1---4

The tendency of proteins of the same/different age classes to form motifs across different PINs (Table S2, S3, S4, S5, S6, S7, S8 and S9) ---------------------------------6

Table S10---10

Table S11---11

Table S12---13

Table S13---14

Table S14---17

Table S15---17

Table S16---18

Results under 10 age classes based on the PIN of DIP_YEAST_CORE (Table S17, S18, S19, S20, S21, S22, S23 and S24)--19

Results under 3 age classes based on the PIN of DIP_YEAST_CORE (Table S25, S26, S27, S28, S29, S30, S31 and S32)---24

Results based on the PIN of DIP_YEAST_CORE when removing the ribosomal proteins (Table S33, S34, S35, S36, S37, S38, S39 and S40)----------------------------29

Figure S1---34

Figure S2---35

References--36

Supplementary Results

The impact of different age classifications

Taking the classification of yeast proteins for example, we group ten time points on the path into 5 age classes mainly based on taxonomy [1-3]. That is, we merge the time points on the path into one age group, whose corresponding extant child species are of close phylogenetic relationship (e.g. we merge node Fungi, node Dikarya, node Ascomycota, node Saccharomyceta, node Saccharomycetales, node Saccharomycetaceae into one age group (age=2), because the five nodes' corresponding extant child species all belong to fungi (Figure 1 in the main document).). We think the taxonomy-based method can help us make time intervals of each age group as even as possible. Of course, it is ideal to merge the time points whose "real emergence ages" are historically close into one age group. However it is unrealistic to obtain enough "real emergence age" information of corresponding ancestral organisms which is often obtained by fossils. Although the taxonomy-based method is rough, in practice we think it is a receivable, approximate method. In addition, the classification can emphasize the representative time points during evolution and thus can contribute to the discovery of natural laws.

Actually we find that all the conclusions in our paper keep unchanged across different classifications of age groups (Table S17, S18, S19, S20, S21, S22, S23 and S24 for 10 age classes; Table S25, S26, S27, S28, S29, S30, S31 and S32 for 3 age classes):

1) In the current PIN, the proteins of the same age class tend to form motifs, while the ones of different age classes tend to avoiding forming motifs (Table S18 and S26).
2) The age homogeneity of motif constituents is affected by their topologies and biological functions (Table S19, S20, S27 and S28).
3) The constituents within the age-homogeneous motifs tend to be densely interconnected, co-evolve and share the same functions, and these motifs tend to be within protein complexes (Table S21, S22, S23, S24, S29, S30, S31 and S32).

Supplementary Methods

Age classification of human proteins

Human proteins are classified into seven age classes based on taxonomy [4] (Figure 1 in the main document): age 1 (human class), proteins that are only found in *H. sapiens*; age 2 (mammalia class), proteins that emerged before the split of *H. sapiens* and other mammalia (node Homininae, node Euarchontoglires, node Eutheria, node Theria, node Mammalia); age 3 (chordata class), proteins that appeared before the split of mammalia and other chordata (node Amniota, node Euteleostomi, node Chordata); age 4 (bilateria class), proteins that emerged before the split of chordata and other bilateria (node Coelomata, node Bilateria); age 5 (fungi/metazoa class), proteins that appeared before the split of bilateria and other fungi/metazoa (node Eumetazoa, node Metazoa and node Fungi/Metazoa group); age 6 (eukaryota class), proteins that evolved before the radiation of eukaryota (node Eukaryota); age 7 (cellular organisms class), proteins that emerged in the common ancestor of three domains of tree of life (node Cellular organisms).

Supplementary Tables and Figures

Table S1 The tendency of proteins of the same/different age classes to form 5-motifs in the PIN of DIP_YEAST_CORE.

5-motif	Empirical *P*-value [a]						
	#5 [b]	#4-1	#3-2	#3-1-1	#2-2-1	#2-1-1-1	#1-1-1-1-1
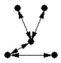	$<10^{-3}$	$<10^{-3}$	0.872	$(<10^{-3})$	$(<10^{-3})$	$(<10^{-3})$	$(<10^{-3})$
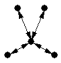	0.018	0.118	0.103	$(<10^{-3})$	$(<10^{-3})$	$(<10^{-3})$	(0.002)
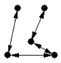	$<10^{-3}$	$<10^{-3}$	0.992	$(<10^{-3})$	$(<10^{-3})$	$(<10^{-3})$	$(<10^{-3})$
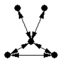	$<10^{-3}$	$<10^{-3}$	0.757	$(<10^{-3})$	$(<10^{-3})$	$(<10^{-3})$	$(<10^{-3})$
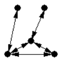	$<10^{-3}$	$<10^{-3}$	1.000	$(<10^{-3})$	$(<10^{-3})$	$(<10^{-3})$	$(<10^{-3})$
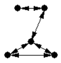	$<10^{-3}$	$<10^{-3}$	1.000	$(<10^{-3})$	$(<10^{-3})$	$(<10^{-3})$	$(<10^{-3})$
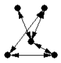	$<10^{-3}$	0.009	0.985	$(<10^{-3})$	$(<10^{-3})$	$(<10^{-3})$	$(<10^{-3})$
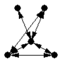	$<10^{-3}$	$<10^{-3}$	0.996	$(<10^{-3})$	$(<10^{-3})$	$(<10^{-3})$	$(<10^{-3})$
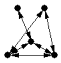	$<10^{-3}$	$<10^{-3}$	0.999	$(<10^{-3})$	$(<10^{-3})$	$(<10^{-3})$	$(<10^{-3})$
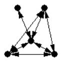	$<10^{-3}$	$<10^{-3}$	1.000	$(<10^{-3})$	$(<10^{-3})$	$(<10^{-3})$	$(<10^{-3})$
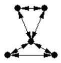	$<10^{-3}$	0.001	1.000	$(<10^{-3})$	$(<10^{-3})$	$(<10^{-3})$	$(<10^{-3})$
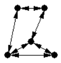	$<10^{-3}$	0.004	0.887	$(<10^{-3})$	$(<10^{-3})$	$(<10^{-3})$	$(<10^{-3})$
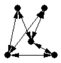	0.005	0.137	0.995	$(<10^{-3})$	$(<10^{-3})$	$(<10^{-3})$	(0.002)
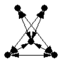	$<10^{-3}$	$<10^{-3}$	0.990	$(<10^{-3})$	$(<10^{-3})$	$(<10^{-3})$	$(<10^{-3})$
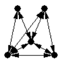	$<10^{-3}$	0.013	0.998	$(<10^{-3})$	$(<10^{-3})$	$(<10^{-3})$	$(<10^{-3})$
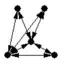	$<10^{-3}$	0.010	0.991	$(<10^{-3})$	$(<10^{-3})$	$(<10^{-3})$	$(<10^{-3})$
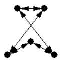	$<10^{-3}$	$<10^{-3}$	0.994	$(<10^{-3})$	$(<10^{-3})$	$(<10^{-3})$	$(<10^{-3})$
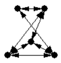	0.001	0.077	0.797	$(<10^{-3})$	$(<10^{-3})$	$(<10^{-3})$	$(<10^{-3})$

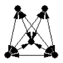	$<10^{-3}$	0.045	0.998	($<10^{-3}$)	($<10^{-3}$)	($<10^{-3}$)	($<10^{-3}$)
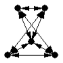	$<10^{-3}$	0.081	0.710	($<10^{-3}$)	($<10^{-3}$)	($<10^{-3}$)	($<10^{-3}$)
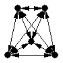	$<10^{-3}$	0.114	1.000	($<10^{-3}$)	($<10^{-3}$)	($<10^{-3}$)	($<10^{-3}$)

[a] For #5, #4-1 and #3-2, upper-tailed *P*-values (enrichment) are listed, and for the other evolutionary motif modes, lower-tailed *P*-values (depletion) in the parentheses are listed. We construct 1000 random networks to evaluate the empirical *P*-values. [b] Labels for evolutionary motif modes. Please refer to Figure 2 in the main document for the definition of the evolutionary motif modes.

The tendency of proteins of the same/different age classes to form motifs across different PINs (Table S2, S3, S4, S5, S6, S7, S8 and S9)

Table S2 Age distribution of proteins in the PIN of DIP_YEAST

Age [a]	Number of proteins
1	520
2	1645
3	546
4	1328
5	512
sum	4551

[a] Please refer to Figure 1 in main document for the age assignment of yeast proteins .

Table S3 Interconnection tendency of proteins of the same/different age classes in the PIN of DIP_YEAST

	Empirical *P*-value [a]				
Binary interaction		#2 [b]		#1-1	
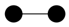		**$<10^{-3}$**		**($<10^{-3}$)**	
3-motif	#3		#2-1		#1-1-1
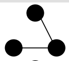	**$<10^{-3}$**		**$<10^{-3}$**		**($<10^{-3}$)**
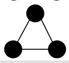	**$<10^{-3}$**		0.223		**($<10^{-3}$)**
4-motif	#4	#3-1	#2-2	#2-1-1	#1-1-1-1
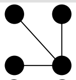	0.076	**0.011**	**$<10^{-3}$**	**(0.005)**	**(0.001)**
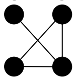	**$<10^{-3}$**	**$<10^{-3}$**	**0.005**	**($<10^{-3}$)**	**($<10^{-3}$)**
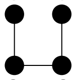	**$<10^{-3}$**	**$<10^{-3}$**	**$<10^{-3}$**	**($<10^{-3}$)**	**($<10^{-3}$)**
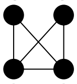	**$<10^{-3}$**	**$<10^{-3}$**	**0.020**	**($<10^{-3}$)**	**($<10^{-3}$)**
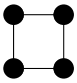	**0.002**	**0.003**	**0.007**	**(0.001)**	**($<10^{-3}$)**
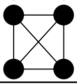	**$<10^{-3}$**	**$<10^{-3}$**	0.136	**($<10^{-3}$)**	**($<10^{-3}$)**

[a] For #2, #3, #2-1, #4, #3-1 and #2-2, upper-tailed *P*-values (enrichment) are listed, and for the other evolutionary motif modes, lower-tailed *P*-values (depletion) in the parentheses are listed. Those lower than 0.05 are highlighted in bold. The empirical *P*-values are computed based on 1000 random networks. [b] Labels for evolutionary motif modes (Figure 2 in the main document).

Table S4 Age distribution of proteins in the PIN of YEAST_HC

Age [a]	Number of proteins
1	116
2	1237
3	412
4	1175
5	324
sum	3264

[a] Please refer to Figure 1 in main document for the age assignment of yeast proteins .

Table S5 Interconnection tendency of proteins of the same/different age classes in the PIN of YEAST_HC

	Empirical *P*-value [a]				
Binary interaction	#2 [a]			#1-1	
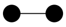	**$<10^{-3}$**			**($<10^{-3}$)**	
3-motif	#3		#2-1	#1-1-1	
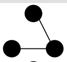	**$<10^{-3}$**		0.117	**($<10^{-3}$)**	
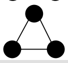	**$<10^{-3}$**		0.993	**($<10^{-3}$)**	
4-motif	#4	#3-1	#2-2	#2-1-1	#1-1-1-1
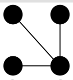	0.388	0.277	0.077	(0.241)	(0.220)
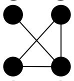	**$<10^{-3}$**	**$<10^{-3}$**	0.811	**($<10^{-3}$)**	**($<10^{-3}$)**
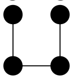	**$<10^{-3}$**	**$<10^{-3}$**	0.512	**($<10^{-3}$)**	**($<10^{-3}$)**
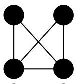	**$<10^{-3}$**	**$<10^{-3}$**	0.209	**($<10^{-3}$)**	**($<10^{-3}$)**
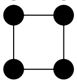	**$<10^{-3}$**	**$<10^{-3}$**	0.959	**($<10^{-3}$)**	**($<10^{-3}$)**
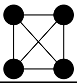	**$<10^{-3}$**	**$<10^{-3}$**	0.544	**($<10^{-3}$)**	**($<10^{-3}$)**

[a] Please refer to the footnotes of Table S3.

Table S6 Age distribution of proteins in the PIN of HPRD_HUMAN_ALL

Age [a]	Number of proteins
1	19
2	559
3	1916
4	298
5	2498
6	1321
7	308
sum	6919

[a] Please refer to Supplementary Methods for the age classification of human proteins

Table S7 Interconnection tendency of proteins of the same/different age classes in the PIN of HPRD_HUMAN_ALL

	Empirical *P*-value [a]				
Binary interaction	#2 [a]			#1-1	
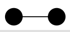	**$<10^{-3}$**			**($<10^{-3}$)**	
3-motif	#3		#2-1	#1-1-1	
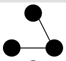	**$<10^{-3}$**		**0.001**	**($<10^{-3}$)**	
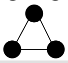	**$<10^{-3}$**		**$<10^{-3}$**	**($<10^{-3}$)**	
4-motif	#4	#3-1	#2-2	#2-1-1	#1-1-1-1
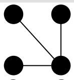	**0.007**	**0.013**	0.113	**(0.032)**	**(0.008)**
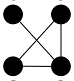	**$<10^{-3}$**	**0.001**	0.122	**(0.001)**	**(0.001)**
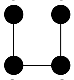	**$<10^{-3}$**	**0.001**	**0.047**	**(0.001)**	**($<10^{-3}$)**
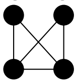	**$<10^{-3}$**	**$<10^{-3}$**	0.084	**($<10^{-3}$)**	**($<10^{-3}$)**
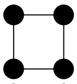	**$<10^{-3}$**	**$<10^{-3}$**	**0.001**	**($<10^{-3}$)**	**($<10^{-3}$)**
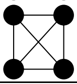	**$<10^{-3}$**	**$<10^{-3}$**	0.173	**($<10^{-3}$)**	**($<10^{-3}$)**

[a] Please refer to the footnotes of Table S3.

Table S8 Age distribution of proteins in the PIN of HPRD_HUMAN_HIGH

Age [a]	Number of proteins
1	15
2	435
3	1651
4	253
5	2050
6	1080
7	220
sum	5704

[a] Please refer to Supplementary Methods for the age classification of human proteins

Table S9 Interconnection tendency of proteins of the same/different age classes in the PIN of HPRD_HUMAN_HIGH

	Empirical *P*-value [a]				
Binary interaction	#2 [a]		#1-1		
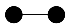	**<10^{-3}**		**(<10^{-3})**		
3-motif	#3		#2-1		#1-1-1
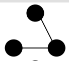	**<10^{-3}**		**0.005**		**(<10^{-3})**
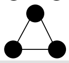	**<10^{-3}**		**0.002**		**(<10^{-3})**
4-motif	#4	#3-1	#2-2	#2-1-1	#1-1-1-1
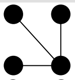	**0.008**	**0.023**	0.196	**(0.042)**	**(0.013)**
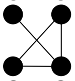	**<10^{-3}**	**0.003**	0.253	**(0.002)**	**(0.010)**
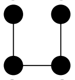	**<10^{-3}**	**0.001**	0.101	**(0.001)**	**(0.003)**
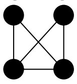	**<10^{-3}**	**0.001**	0.183	**(0.001)**	**(<10^{-3})**
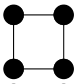	**<10^{-3}**	**0.001**	**0.003**	**(0.001)**	**(<10^{-3})**
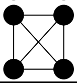	**<10^{-3}**	**0.001**	0.278	**(<10^{-3})**	**(0.002)**

[a] Please refer to the footnotes of Table S3.

Table S10 Constraints of topologies on the co-origins of motif constituents for 5-motifs in the PIN of DIP_YEAST_CORE.

5-motif	The total number	Age homogeneity rate (%) [a]		Age homogeneity ratio [b]
		Real network	Average of 1000 random networks	
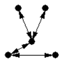	5766292	6.45	1.33	4.85
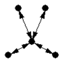	4285494	3.82	1.31	2.91
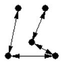	2391633	8.83	1.34	6.61
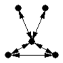	504884	11.37	1.32	8.64
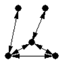	399622	13.39	1.33	10.05
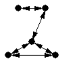	341537	15.64	1.33	11.78
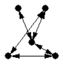	181750	8.59	1.34	6.41
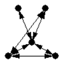	97078	21.97	1.34	16.34
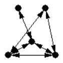	71141	18.79	1.34	14.01
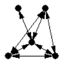	27356	30.36	1.32	23.08
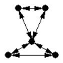	12848	29.30	1.33	21.99
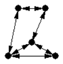	11406	17.20	1.34	12.83
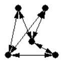	11021	7.26	1.33	5.45
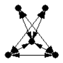	9125	31.93	1.34	23.90
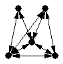	6372	40.32	1.33	30.27
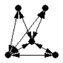	6027	26.23	1.38	19.03
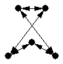	4237	12.20	1.33	9.19
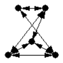	4179	14.69	1.37	10.74
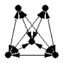	1808	45.46	1.34	33.88

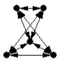	871	28.36	1.37	20.68
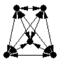	632	40.82	1.28	31.87

[a] Age homogeneity rate is referred to as the fraction of motifs whose constituents are of the same age class. [b] Age homogeneity ratio is defined as the ratio of the age homogeneity rate of the real network to its random expectation which is calculated as the average age homogeneity rate of the 1000 random networks (the fourth column).

Table S11 The correlation between the topological saturation and age homogeneity for motifs. Here the topological saturation of motifs is measured by the number of edges within the motifs. Spearman's rank correlation analysis is used. The result is based on the PIN of DIP_YEAST_CORE. (A) 3-motif. r=-0.09, $P<10^{-4}$. (B) 4-motif. r=-0.11, $P<10^{-4}$. (C) 5-motif. r=-0.11, $P<10^{-4}$.

3-motif	The number of motifs			
	Evolutionary motif mode: [a]	#3	#2-1	#1-1-1
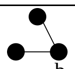 (2) [b]		11420	29486	9630
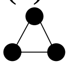 (3)		1037	1293	276

(A)

[a] Please refer to Figure 2 in main document for the definition of evolutionary motif modes. [b] The number in the parentheses denotes the number of edges within the motif, which can simply measure the topological saturation of the motif.

.

4-motif	The number of motifs					
	Evolutionary motif mode:	#4	#3-1	#2-2	#2-1-1	#1-1-1-1
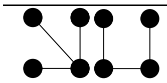 (3)		85680	264599	115834	241105	25089
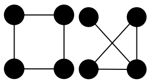 (4)		14466	23635	7380	13928	1029
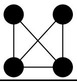		2018	2035	657	994	44

(5) 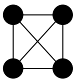 (6)	525	448	137	199	6

(B)

5-motif		The number of motifs						
	Evolutionary motif mode:	#5	#4-1	#3-2	#3-1-1	#2-2-1	#2-1-1-1	#1-1-1-1-1
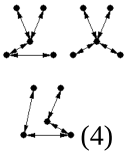 (4)		746585	2799730	2694629	2889412	2251310	1032896	28857
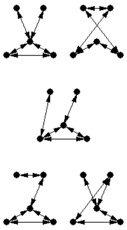 (5)		180485	421588	260841	294283	199297	74286	1250
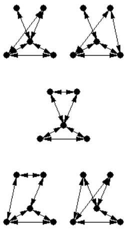 (6)		41224	65621	31939	34408	22950	7245	107
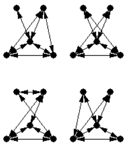 (7)		13414	15882	5924	6285	4290	885	7
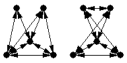 (8)		2816	2121	928	746	555	77	0
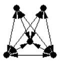 (9)		822	527	196	146	100	17	0
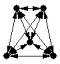 (10)		258	186	85	65	36	2	0

(C)

Table S12 Detailed description for abbreviations of functional categories which are from MIPS/CYGD database [5].

Abbreviation	Detailed description
Metabolism	Metabolism
Energy	Energy
Cell cycle	Cell cycle and DNA processing
Transcription	Transcription
Protein synthesis	Protein synthesis
Protein fate	Protein fate (folding, modification, destination)
Binding protein	Protein with binding function or cofactor requirement (structural or catalytic)
Regulation of metabolism	Regulation of metabolism and protein function
Cellular transport	Cellular transport, transport facilities and transport routes
Signal transduction	Cellular communication/signal transduction mechanism
Cell defense	Cell rescue, defense and virulence
Environment interaction	Interaction with the environment
Cell fate	Cell fate
Cellular components development	Biogenesis of Cellular components development (systemic)
Transposable proteins	Transposable elements, viral and plasmid proteins
Cell differentiation	Cell type differentiation
Unclassified proteins	Unclassified proteins

Table S13 Joint constraints of functions and topologies on co-origins of motif constituents in the PIN of DIP_YEAST_CORE.

Functional category [a]	topology	Total number	Age homogeneity rate (%) [c]		Empirical *P*-value [b]	Age homogeneity ratio [d]
			Real network	Average of 1000 random networks		
Metabolism	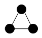	3008	13.5	10.2	0.096	1.32
	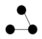	193	31.1	9.9	$<10^{-3}$	3.13
	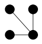	9020	4.1	3.7	0.340	1.11
	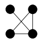	12242	5.0	3.7	0.238	1.35
	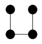	1419	8.9	3.6	0.016	2.46
	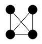	69	8.7	3.8	0.076	2.30
	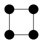	183	20.2	3.6	0.002	5.59
	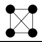	48	29.2	3.5	$<10^{-3}$	8.37
Energy	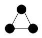	235	8.9	9.9	0.528	0.90
	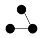	35	17.1	10.1	0.174	1.70
	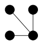	44	2.3	3.7	0.340	0.62
	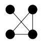	659	1.2	3.5	0.696	0.35
	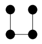	137	1.5	3.6	0.536	0.40
	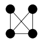	0	-	-	-	-
	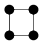	101	5.9	3.3	0.172	1.80
	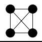	8	0.0	3.8	0.176	0.00
Cell cycle	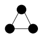	5070	20.5	10.1	$<10^{-3}$	2.04
	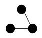	524	27.3	10.1	$<10^{-3}$	2.70
	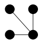	17488	12.0	3.6	$<10^{-3}$	3.32
	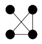	34667	5.8	3.6	0.228	1.59
	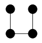	4445	15.7	3.6	$<10^{-3}$	4.36
	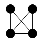	99	17.2	3.6	$<10^{-3}$	4.74
	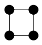	464	16.6	3.6	$<10^{-3}$	4.63
	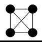	303	12.2	3.8	0.034	3.23
Transcription	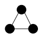	5997	33.5	10.0	$<10^{-3}$	3.34
	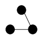	787	37.0	10.0	$<10^{-3}$	3.68
	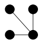	22796	19.6	3.6	$<10^{-3}$	5.43
	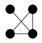	19803	28.1	3.6	$<10^{-3}$	7.84
	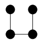	9288	26.3	3.6	$<10^{-3}$	7.30
	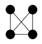	282	26.2	3.5	$<10^{-3}$	7.39
	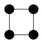	1345	25.2	3.6	$<10^{-3}$	7.00
	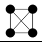	414	23.4	3.6	0.002	6.48
Protein synthesis	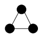	250	32.4	10.2	$<10^{-3}$	3.18
	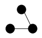	55	43.6	10.2	0.002	4.27
	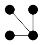	327	16.5	3.8	0.008	4.32
	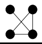	192	21.9	3.6	0.002	6.06

	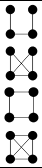	160	18.1	3.7	0.006	4.84
		19	5.3	3.9	0.172	1.35
		52	25.0	3.7	0.006	6.81
		15	40.0	4.2	0.014	9.62
protein fate	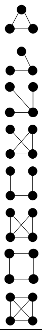	6308	36.3	10.2	$<10^{-3}$	3.57
		865	49.1	10.1	$<10^{-3}$	4.86
		25692	24.4	3.7	$<10^{-3}$	6.64
		21182	26.3	3.7	$<10^{-3}$	7.15
		10764	43.3	3.6	$<10^{-3}$	11.90
		998	16.2	3.8	$<10^{-3}$	4.29
		2188	47.7	3.6	$<10^{-3}$	13.09
		646	53.1	3.6	$<10^{-3}$	14.70
Binding protein	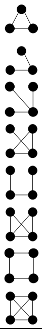	4274	26.5	10.2	$<10^{-3}$	2.60
		481	28.9	10.3	$<10^{-3}$	2.82
		12520	13.9	3.7	$<10^{-3}$	3.80
		11908	21.6	3.7	$<10^{-3}$	5.92
		4169	19.1	3.6	$<10^{-3}$	5.24
		393	9.2	3.8	0.048	2.43
		697	14.8	3.8	$<10^{-3}$	3.93
		218	16.5	3.8	0.014	4.34
Regulation of metabolism	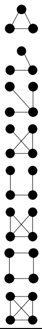	78	26.9	10.5	0.018	2.57
		18	16.7	10.0	0.182	1.67
		49	24.5	3.6	$<10^{-3}$	6.86
		68	10.3	3.9	0.086	2.65
		9	11.1	3.6	0.062	3.05
		1	100.0	2.6	$<10^{-3}$	38.46
		8	0.0	3.5	0.186	0.00
		7	0.0	3.5	0.178	0.00
Cellular transport	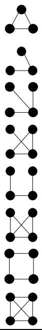	6275	28.3	10.1	$<10^{-3}$	2.79
		536	38.6	10.2	$<10^{-3}$	3.80
		27078	17.7	3.7	$<10^{-3}$	4.82
		23437	15.9	3.7	$<10^{-3}$	4.35
		7336	23.2	3.7	$<10^{-3}$	6.33
		1884	17.7	3.7	$<10^{-3}$	4.74
		1327	26.8	3.7	$<10^{-3}$	7.22
		168	42.3	3.8	$<10^{-3}$	11.16
Signal transduction	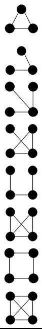	372	13.7	10.4	0.158	1.32
		27	22.2	10.0	0.042	2.22
		791	5.3	3.8	0.204	1.39
		399	4.8	3.8	0.256	1.26
		172	4.1	3.7	0.318	1.10
		18	5.6	4.0	0.154	1.40
		15	6.7	3.5	0.090	1.91
		2	0.0	3.6	0.058	0.00

Cell defense	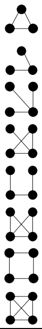	522	18.0	10.1	0.016	1.77
		49	28.6	10.0	0.002	2.85
		967	8.8	3.5	0.012	2.48
		598	6.5	3.6	0.104	1.80
		266	10.5	3.6	0.018	2.90
		14	7.1	3.6	0.112	2.00
		39	7.7	3.6	0.112	2.13
		5	20.0	4.0	0.022	5.05
environment interaction	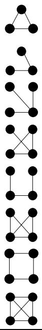	674	16.0	10.1	0.044	1.59
		85	18.8	10.2	0.062	1.85
		1112	5.6	3.6	0.174	1.53
		887	6.9	3.7	0.090	1.88
		320	6.3	3.5	0.132	1.76
		73	15.1	3.7	0.038	4.04
		74	12.2	3.5	0.056	3.48
		20	5.0	4.1	0.120	1.23
Cell fate	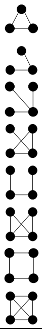	1393	19.3	10.0	0.004	1.93
		112	25.0	10.0	0.006	2.51
		4803	7.7	3.6	0.016	2.15
		3085	8.2	3.5	0.014	2.32
		1349	9.9	3.6	0.022	2.78
		60	6.7	3.6	0.126	1.85
		164	14.6	3.6	0.022	4.06
		23	8.7	3.6	0.088	2.43
Cellular components	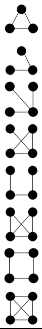	3161	20.1	10.1	0.002	1.99
		360	29.2	10.2	$<10^{-3}$	2.87
		11655	8.8	3.6	0.002	2.42
		8509	8.5	3.6	0.01	2.37
		3523	12.8	3.6	$<10^{-3}$	3.56
		295	5.1	3.7	0.212	1.36
		535	15.3	3.7	$<10^{-3}$	4.12
		130	19.2	3.8	0.006	5.12
Cell differentiation	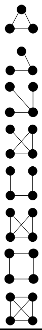	2188	18.1	10.0	0.004	1.81
		190	27.4	10.0	$<10^{-3}$	2.72
		8683	7.6	3.6	0.010	2.12
		5637	7.2	3.5	0.028	2.06
		2390	10.7	3.5	0.004	3.04
		106	7.5	3.5	0.072	2.16
		275	14.5	3.6	$<10^{-3}$	4.04
		49	18.4	3.7	0.016	4.93

[a] Here we list the abbreviations of functional categories and please refer to Table S12 for the details. [b] Here we list the upper-tailed empirical *P*-values (enrichment). The *P*-values lower than 0.05 are highlighted in red. [c] Age homogeneity rate is referred to

as the fraction of motifs whose constituents are of the same age class. [d] Age homogeneity ratio is defined as the ratio of the age homogeneity rate of the real network to its random expectation.

Table S14 Age homogeneity rates of motifs whose constituents share at least one common functional category and the other motifs. The result is based on the PIN of DIP_YEAST_CORE.

	Motifs whose members share at least one common functional category		The other motifs		P-value (Chi-squared test)
	Total number	Age homogeneity rate [a] (%)	Total number	Age homogeneity rate (%)	
Binary interaction	4369	46.2	1242	32.2	$<10^{-4}$
3-motif	28942	28.3	24200	17.6	$<10^{-4}$
4-motif	268821	17.8	530987	10.3	$<10^{-4}$

[a] Age homogeneity rate is referred to as the fraction of motifs whose constituents are of the same age class.

Table S15 The fraction of motifs within protein complexes among the motifs with constituents of the same age class and that among the motifs of different age classes. The motifs are from the PIN of DIP_YEAST_CORE.

	Motifs whose members are of the same age class		Motifs whose members are of different age classes		P-value (Chi-squared test)
	Total number	The fraction of motifs within protein complexes (%)	Total number	The fraction of motifs within protein complexes (%)	
3-motif	12457	6.46	40685	3.25	$<10^{-4}$
4-motif	102689	1.73	697119	0.59	$<10^{-4}$

Table S16 Topology distribution of motifs whose members of the same age class and those of different age classes. The motif topologies are classified based on the topological saturation measured by the number of edges within the motif. (A) 3-motif. (B) 4-motif. Rank sum test is used to measure statistical significance of the difference between topology distributions of the two classes of motifs. $P<10^{-4}$ for both 3-motifs and 4-motifs. Results are based on the PIN of DIP_YEAST_CORE.

	The number of motifs	
	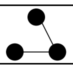	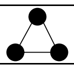
3-motifs whose members are of the same age class	11420	1037
The other 3-motifs	39116	1569

(A)

	The number of motifs			
	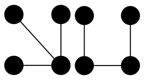	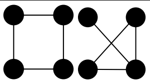	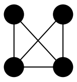	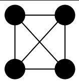
4-motifs whose members are of the same age class	85680	14466	2018	525
The other 4-motifs	646627	45972	3730	790

(B)

Results under 10 age classes based on the PIN of DIP_YEAST_CORE (Table S17, S18, S19, S20, S21, S22, S23 and S24)

Table S17 The age distribution of proteins in the PIN of DIP_YEAST_CORE

Age [a]	Number of proteins
1 (yeast class)	113
2 (Saccharomycetaceae class)	264
3 (Saccharomycetales class)	193
4 (Saccharomyceta class)	53
5 (Ascomycota class)	85
6 (Dikarya class)	286
7 (Fungi class)	15
8 (Fungi/Metazoa group class)	301
9 (Eukaryota class)	967
10 (Cellular organisms class)	268
sum	2545

[a] Here the yeast proteins are classified into ten age classes (Figure 1 in the main document).

Table S18 Interconnection tendency of proteins of the same/different age classes in the PIN of DIP_YEAST_CORE

	Empirical *P*-value [a]				
Binary interaction	#2 [a]		#1-1		
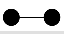	**$<10^{-3}$**		**($<10^{-3}$)**		
3-motif	#3		#2-1		#1-1-1
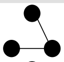	**$<10^{-3}$**		**$<10^{-3}$**		**($<10^{-3}$)**
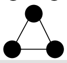	**$<10^{-3}$**		0.217		**($<10^{-3}$)**
4-motif	#4	#3-1	#2-2	#2-1-1	#1-1-1-1
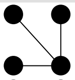	**$<10^{-3}$**	**$<10^{-3}$**	0.074	**($<10^{-3}$)**	**($<10^{-3}$)**
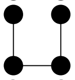	**$<10^{-3}$**	**$<10^{-3}$**	**0.019**	**($<10^{-3}$)**	**($<10^{-3}$)**
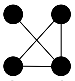	**$<10^{-3}$**	**$<10^{-3}$**	0.385	**($<10^{-3}$)**	**($<10^{-3}$)**
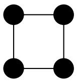	**$<10^{-3}$**	**0.001**	0.256	**($<10^{-3}$)**	**(0.001)**
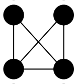	**$<10^{-3}$**	**$<10^{-3}$**	0.728	**($<10^{-3}$)**	**($<10^{-3}$)**
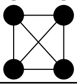	**$<10^{-3}$**	**$<10^{-3}$**	0.620	**($<10^{-3}$)**	**($<10^{-3}$)**

[a] Please refer to the footnotes of Table S3.

Table S19 Constraints of topologies on the co-origins of motif constituents in the PIN of DIP_YEAST_CORE.

motif	The total number	Age homogeneity rate (%) [a]		Age homogeneity ratio [b]
		Real network	Average of 1000 random networks	
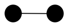	5611	36.59	20.23	1.81
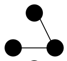	50536	19.78	6.11	3.24
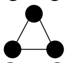	2606	37.53	6.08	6.17
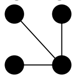	400510	8.99	2.15	4.17
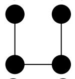	331797	12.89	2.17	5.94
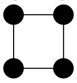	4746	15.07	2.17	6.95
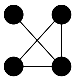	55692	23.61	2.16	10.91
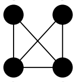	5748	34.19	2.16	15.79
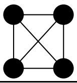	1315	39.39	2.12	18.59

[a] Age homogeneity rate is referred to as the fraction of motifs whose constituents are of the same age class. [b] Age homogeneity ratio is defined as the ratio of the age homogeneity rate of the real network to its random expectation.

Table S20 Constraints of functions on the co-origins of motif constituents in the PIN of DIP_YEAST_CORE.

Functional Category [a]	The total number	Age homogeneity rate (%) [c]		Empirical *P*-value [b]	Age homogeneity ratio [d]
		Real network	Average of 1000 random networks		
3-motif					
Metabolism	3201	12.65	6.13	**0.012**	2.07
Energy	270	10.00	6.31	0.185	1.58
Cell cycle	5594	19.45	6.09	**<10^{-3}**	3.19
Transcription	6784	30.84	6.06	**<10^{-3}**	5.09
Protein synthesis	305	34.43	6.33	**0.001**	5.44
protein fate	7173	37.00	6.16	**<10^{-3}**	6.01
Binding protein	4755	24.67	6.10	**<10^{-3}**	4.05
Regulation of metabolism	96	18.75	5.98	**0.027**	3.13
Cellular transport	6811	25.41	6.08	**<10^{-3}**	4.18
Signal transduction	399	8.52	6.24	0.205	1.37
Cell defense	571	16.99	6.16	**0.003**	2.76
environment interaction	759	9.35	6.12	0.119	1.53
Cell fate	1505	10.43	6.05	0.062	1.73
Cellular components	3521	14.91	6.04	**<10^{-3}**	2.47
Cell differentiation	2378	11.77	6.04	**0.022**	1.95
4-motif					
Metabolism	22981	4.73	2.17	0.063	2.18
Energy	949	1.79	2.31	0.373	0.78
Cell cycle	57466	8.33	2.15	**0.011**	3.87
Transcription	53928	22.69	2.14	**<10^{-3}**	10.62
Protein synthesis	765	18.95	2.37	**0.002**	8.01
protein fate	61470	29.18	2.20	**<10^{-3}**	13.24
Binding protein	29905	17.09	2.16	**<10^{-3}**	7.93
Regulation of metabolism	142	10.56	2.17	**0.036**	4.87
Cellular transport	61230	15.94	2.16	**<10^{-3}**	7.37
Signal transduction	1397	2.15	2.25	0.366	0.95
Cell defense	1889	7.89	2.19	**0.017**	3.60
environment interaction	2486	2.65	2.18	0.271	1.22
Cell fate	9484	3.46	2.13	0.177	1.62
Cellular components	24647	6.78	2.12	**0.006**	3.19
Cell differentiation	17140	4.82	2.11	0.059	2.28

[a] Here we list the abbreviations of functional categories and please refer to Table S12 for the details. [b] Here we list upper-tailed empirical *P*-values (enrichment). The *P*-values lower than 0.05 are highlighted in bold. [c] Age homogeneity rate is referred to

as the fraction of motifs whose constituents are of the same age class. [d] Age homogeneity ratio is defined as the ratio of the age homogeneity rate of the real network to its random expectation.

Table S21 Average evolutionary rate difference of protein pairs within motifs whose members are of the same age class and within those of different age classes. The result is based on the PIN of DIP_YEAST_CORE.

	Average evolutionary rate difference of protein pairs		*P*-value (Rank sum test)
	motifs whose constituents are of the same age class	The other motifs	
3-motifs	0.06	0.08	$<10^{-4}$
4-motifs	0.06	0.08	$<10^{-4}$

Table S22 Functional homogeneity rates of motifs whose constituents are of the same age class and those of different age classes. The result is based on the PIN of DIP_YEAST_CORE.

	Motifs whose members are of the same age class		The other motifs		*P*-value (Chi-squared test)
	Total number	Functional homogeneity rate [a] (%)	Total number	Functional homogeneity rate (%)	
Binary interaction	2053	85.2	3558	73.6	$<10^{-4}$
3-motif	10975	67.1	42167	51.2	$<10^{-4}$
4-motif	95110	47.2	704698	31.8	$<10^{-4}$

[a] The "functional homogeneity rate" is calculated as the number of motifs whose members share at least one common functional category divided by the total number of motifs.

Table S23 Topology distribution of motifs whose members of the same age class and those of different age classes. The motif topologies are classified based on the topological saturation measured by the number of edges within the motif. The result is based on the PIN of DIP_YEAST_CORE. (A) 3-motif. (B) 4-motif. Rank sum test, $P<10^{-4}$ for both 3-motif and 4-motif.

	The number of motifs	
	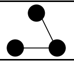	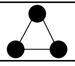
3-motifs whose members are of the same age class	9997	978
The other 3-motifs	40539	1628

(A)

	The number of motifs			
	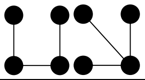	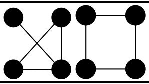	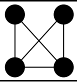	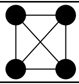
4-motifs whose members are of the same age class	78764	13863	1965	518
The other 4-motifs	653543	46575	3783	797

(B)

Table S24 The fraction of motifs within protein complexes among the motifs with constituents of the same age class and that among the motifs of different age classes. The result is based on the PIN of DIP_YEAST_CORE.

	Motifs whose members are of the same age class		The other motifs		*P*-value (Chi-squared test)
	Total number	The fraction of motifs within protein complexes (%)	Total number	The fraction of motifs within protein complexes (%)	
3-motif	10975	6.72	42167	3.29	$<10^{-4}$
4-motif	95110	1.74	704698	0.60	$<10^{-4}$

Results under 3 age classes based on the PIN of DIP_YEAST_CORE (Table S25, S26, S27, S28, S29, S30, S31 and S32)

Table S25 The age distribution of proteins in the PIN of DIP_YEAST_CORE

Age [a]	The number of proteins
1	1009
2	1268
3	268
sum	2545

[a] Here we classify the yeast proteins into three age classes. Age 1 (fungi class: node Fungi, node Dikarya, node Ascomycota, node Saccharomyceta, node Saccharomycetales, node Saccharomycetaceae and yeast); Age 2 (eukaryota class: node Fungi/Metazoa group and node Eukaryota); Age 3 (cellular organisms class: node Cellular organisms) (Figure 1 in the main document).

Table S26 Interconnection tendency of proteins of the same/different age classes in the PIN of DIP_YEAST_CORE.

	Empirical P-value [a]				
Binary interaction	#2 [a]			#1-1	
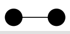	**$<10^{-3}$**			**($<10^{-3}$)**	
3-motif	#3		#2-1		#1-1-1
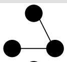	**$<10^{-3}$**		**1.000**		**($<10^{-3}$)**
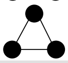	**$<10^{-3}$**		**1.000**		**($<10^{-3}$)**
4-motif	#4	#3-1	#2-2	#2-1-1	#1-1-1-1
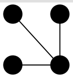	**$<10^{-3}$**	**$<10^{-3}$**	0.815	**($<10^{-3}$)**	-
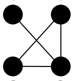	**$<10^{-3}$**	**0.031**	1.000	**($<10^{-3}$)**	-
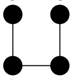	**$<10^{-3}$**	**$<10^{-3}$**	1.000	**($<10^{-3}$)**	-
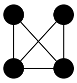	**$<10^{-3}$**	0.914	1.000	**($<10^{-3}$)**	-
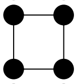	**$<10^{-3}$**	**0.001**	0.991	**($<10^{-3}$)**	-
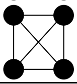	**$<10^{-3}$**	0.978	1.000	**($<10^{-3}$)**	-

[a] Please refer to the footnotes of Table S3.

Table S27 Constraints of topologies on the co-origins of motif constituents in the PIN of DIP_YEAST_CORE.

motif	The total number	Age homogeneity rate (%) [a]		Age homogeneity ratio [b]
		Real network	Average of 1000 random networks	
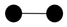	5611	54.23	41.66	1.30
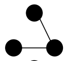	50536	33.26	18.74	1.78
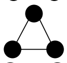	2606	48.20	18.75	2.57
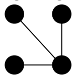	400510	17.29	8.65	2.00
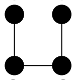	331797	23.29	8.67	2.69
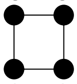	4746	27.24	8.68	3.14
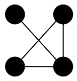	55692	32.79	8.67	3.78
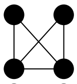	5748	42.07	8.71	4.83
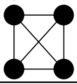	1315	45.40	8.71	5.21

[a] Age homogeneity rate is referred to as the fraction of motifs whose constituents are of the same age class. [b] Age homogeneity ratio is defined as the ratio of the age homogeneity rate of the real network to its random expectation.

Table S28 Constraints of functions on the co-origins of motif constituents in the PIN of DIP_YEAST_CORE.

Functional Category [a]	The total number	Age homogeneity rate (%) [c]		Empirical *P*-value [b]	Age homogeneity ratio [d]
		Real network	Average of 1000 random networks		
3-motif					
Metabolism	3201	40.67	18.75	**0.006**	2.17
Energy	270	36.78	18.44	0.620	1.99
Cell cycle	5594	15.56	18.78	**<10^{-3}**	0.83
Transcription	6784	36.04	18.63	**<10^{-3}**	1.93
Protein synthesis	305	33.33	18.80	**<10^{-3}**	1.77
protein fate	7173	22.31	18.92	**<10^{-3}**	1.18
Binding protein	4755	37.22	18.70	**<10^{-3}**	1.99
Regulation of metabolism	96	27.27	18.73	**0.049**	1.46
Cellular transport	6811	32.43	18.67	**<10^{-3}**	1.74
Signal transduction	399	66.67	18.88	0.215	3.53
Cell defense	571	47.21	18.78	**0.001**	2.51
environment interaction	759	48.75	18.58	0.106	2.62
Cell fate	1505	42.18	18.68	**0.002**	2.26
Cellular components	3521	30.69	18.75	**<10^{-3}**	1.64
Cell differentiation	2378	34.69	18.66	**<10^{-3}**	1.86
4-motif					
Metabolism	22981	30.50	8.68	**0.033**	3.51
Energy	949	23.24	8.45	0.680	2.75
Cell cycle	57466	5.06	8.73	0.085	0.58
Transcription	53928	24.10	8.57	**<10^{-3}**	2.81
Protein synthesis	765	21.83	8.77	**0.004**	2.49
protein fate	61470	8.80	8.80	**<10^{-3}**	1.00
Binding protein	29905	26.91	8.61	**<10^{-3}**	3.13
Regulation of metabolism	142	14.48	8.76	0.058	1.65
Cellular transport	61230	18.08	8.62	**<10^{-3}**	2.10
Signal transduction	1397	100.00	8.78	0.415	11.39
Cell defense	1889	32.94	8.64	**0.001**	3.81
environment interaction	2486	39.11	8.50	0.250	4.60
Cell fate	9484	29.11	8.59	**0.010**	3.39
Cellular components	24647	14.08	8.63	**<10^{-3}**	1.63
Cell differentiation	17140	21.58	8.57	**<10^{-3}**	2.52

[a] Here we list the abbreviations of functional categories and please refer to Table S12 for the details. [b] Here we list upper-tailed empirical *P*-values (enrichment). The *P*-values lower than 0.05 are highlighted in bold. [c] Age homogeneity rate is referred to

as the fraction of motifs whose constituents are of the same age class. [d] Age homogeneity ratio is defined as the ratio of the age homogeneity rate of the real network to its random expectation.

Table S29 Average evolutionary rate difference of protein pairs within motifs whose members are of the same age class and within those of different age classes. The result is based on the PIN of DIP_YEAST_CORE.

	Average evolutionary rate difference of protein pairs		*P*-value (Rank sum test)
	motifs whose constituents are of the same age class	The other motifs	
3-motifs	0.06	0.08	$<10^{-4}$
4-motifs	0.06	0.08	$<10^{-4}$

Table S30 Functional homogeneity rates of motifs whose constituents are of the same age class and those of different age classes. The result is based on the PIN of DIP_YEAST_CORE.

	Motifs whose members are of the same age class		The other motifs		*P*-value (Chi-squared test)
	Total number	Functional homogeneity rate [a] (%)	Total number	Functional homogeneity rate (%)	
Binary interaction	3043	81.4	2568	73.7	$<10^{-4}$
3-motif	18065	61.8	35077	50.7	$<10^{-4}$
4-motif	169122	42.0	630686	31.4	$<10^{-4}$

[a] The "functional homogeneity rate" is calculated as the number of motifs whose members share at least one common functional category divided by the total number of motifs.

Table S31 Topology distribution of motifs whose members of the same age class and those of different age classes. The motif topologies are classified based on the topological saturation measured by the number of edges within the motif. The result is based on the PIN of DIP_YEAST_CORE. (A) 3-motif. (B) 4-motif. Rank sum test, $P<10^{-4}$ for both 3-motif and 4-motif.

	The number of motifs	
	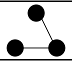	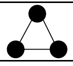
3-motifs whose members are of the same age class	16809	1256
The other 3-motifs	33727	1350

(A)

	The number of motifs			
	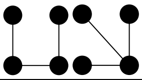	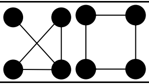	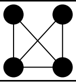	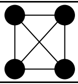
4-motifs whose members are of the same age class	146550	19557	2418	597
The other 4-motifs	585757	40881	3330	718

(B)

Table S32 The fraction of motifs within protein complexes among the motifs with constituents of the same age class and that among the motifs of different age classes.

	Motifs whose members are of the same age		Motifs whose members are of different age classes		*P*-value (Chi-squared test)
	Total number	The fraction of motifs within protein complexes (%)	Total number	The fraction of motifs within protein complexes (%)	
3-motif	18065	5.08	35077	3.44	$<10^{-4}$
4-motif	169122	1.18	630686	0.62	$<10^{-4}$

Results based on the PIN of DIP_YEAST_CORE when removing the ribosomal proteins (Table S33, S34, S35, S36, S37, S38, S39 and S40)

Table S33 The age distribution of proteins in the PIN of DIP_YEAST_CORE.

Age [a]	Number of proteins
1	113
2	884
3	296
4	945
5	243
sum	2481

[a] Please refer to Figure 1 in main document for the age assignment of yeast proteins .

Table S34 Interconnection tendency of proteins of the same/different age classes in the PIN of DIP_YEAST_CORE.

	Empirical *P*-value [a]				
Binary interaction	#2 [a]			#1-1	
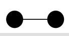	**$<10^{-3}$**			**$<10^{-3}$**	
3-motif	#3		#2-1	#1-1-1	
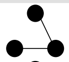	**$<10^{-3}$**		0.520	**($<10^{-3}$)**	
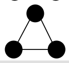	**$<10^{-3}$**		1.000	**($<10^{-3}$)**	
4-motif	#4	#3-1	#2-2	#2-1-1	#1-1-1-1
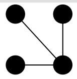	**$<10^{-3}$**	**$<10^{-3}$**	0.100	**($<10^{-3}$)**	**($<10^{-3}$)**
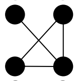	**$<10^{-3}$**	**$<10^{-3}$**	0.999	**($<10^{-3}$)**	**($<10^{-3}$)**
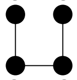	**$<10^{-3}$**	**$<10^{-3}$**	0.783	**($<10^{-3}$)**	**($<10^{-3}$)**
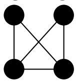	**$<10^{-3}$**	**0.001**	0.995	**($<10^{-3}$)**	**($<10^{-3}$)**
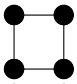	**$<10^{-3}$**	**$<10^{-3}$**	0.775	**($<10^{-3}$)**	**($<10^{-3}$)**
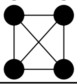	**$<10^{-3}$**	**0.024**	0.987	**($<10^{-3}$)**	**($<10^{-3}$)**

[a] Please refer to the footnotes of Table S3

Table S35 Constraints of topologies on the co-origins of motif constituents in the PIN of DIP_YEAST_CORE.

motif	The total number	Age homogeneity rate (%) [a]		Age homogeneity ratio [b]
		Real network	Average of 1000 random networks	
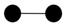	5503	43.2	29.8	1.45
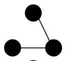	49257	22.5	10.3	2.18
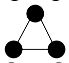	2572	39.7	10.3	3.85
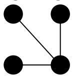	387375	9.9	3.7	2.68
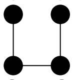	321951	13.6	3.7	3.68
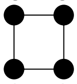	4687	16.7	3.8	4.39
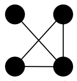	54153	24.4	3.7	6.59
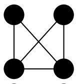	5618	35.1	3.8	9.24
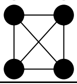	1307	40.0	3.8	10.53

[a] Age homogeneity rate is referred to as the fraction of motifs whose constituents are of the same age class. [b] Age homogeneity ratio is defined as the ratio of the age homogeneity rate of the real network to its random expectation.

Table S36 Constraints of functions on the co-origins of motif constituents in the PIN of DIP_YEAST_CORE.

Functional Category [a]	The total number	Age homogeneity rate (%) [c]		Empirical *P*-value [b]	Age homogeneity ratio [d]
		Real network	Average of 10000 random networks		
3-motif					
Metabolism	3191	14.6	10.3	**0.037**	1.42
Energy	270	10.0	10.3	0.451	0.97
Cell cycle	5594	21.1	10.3	**$<10^{-3}$**	2.05
Transcription	6502	33.7	10.4	**$<10^{-3}$**	3.24
Protein synthesis	213	28.2	10.4	**0.004**	2.72
protein fate	7113	38.1	10.2	**$<10^{-3}$**	3.72
Binding protein	4645	26.4	10.2	**$<10^{-3}$**	2.58
Regulation of metabolism	96	25.0	10.4	**0.016**	2.41
Cellular transport	6811	29.1	10.2	**$<10^{-3}$**	2.85
Signal transduction	399	14.3	10.4	0.144	1.37
Cell defense	571	18.9	10.2	**0.01**	1.85
environment interaction	759	16.3	10.3	**0.024**	1.58
Cell fate	1505	19.7	10.3	**0.006**	1.91
Cellular components	3517	21.0	10.2	**$<10^{-3}$**	2.05
Cell differentiation	2374	18.9	10.2	**0.003**	1.86
4-motif					
Metabolism	22896	5.1	3.8	0.176	1.34
Energy	949	1.8	4.0	0.624	0.45
Cell cycle	57466	8.6	4.7	**0.023**	1.83
Transcription	51220	23.7	4.9	**$<10^{-3}$**	4.86
Protein synthesis	467	12.6	5.0	**0.020**	2.55
protein fate	60491	29.8	5.1	**$<10^{-3}$**	5.86
Binding protein	29097	17.6	5.3	**$<10^{-3}$**	3.33
Regulation of metabolism	142	14.8	5.6	**0.026**	2.65
Cellular transport	61230	17.9	5.7	**$<10^{-3}$**	3.16
Signal transduction	1397	5.0	6.8	0.239	0.74
Cell defense	1889	8.3	6.8	**0.025**	1.22
environment interaction	2486	6.6	7.1	0.072	0.92
Cell fate	9484	8.3	7.7	**0.020**	1.07
Cellular components	24630	9.4	7.9	**0.002**	1.19
Cell differentiation	17115	8.1	7.9	**0.014**	1.01

[a] Here we list the abbreviations of functional categories and please refer to Table S12 for the details. [b] Here we list upper-tailed empirical *P*-values (enrichment). The *P*-values lower than 0.05 are highlighted in bold. [c] Age homogeneity rate is referred to

as the fraction of motifs whose constituents are of the same age class. [d] Age homogeneity ratio is defined as the ratio of the age homogeneity rate of the real network to its random expectation.

Table S37 Average evolutionary rate difference of protein pairs within motifs whose members are of the same age class and within those of different age classes. The result is based on the PIN of DIP_YEAST_CORE.

	Average evolutionary rate difference of protein pairs		*P*-value (Rank sum test)
	motifs whose constituents are of the same age class	The other motifs	
3-motifs	0.06	0.08	$<10^{-4}$
4-motifs	0.06	0.08	$<10^{-4}$

Table S38 Functional homogeneity rates of motifs whose constituents are of the same age class and those of different age classes. The result is based on the PIN of DIP_YEAST_CORE.

	Motifs whose members are of the same age class		The other motifs		*P*-value (Chi-squared test)
	Total number	Functional homogeneity rate [a] (%)	Total number	Functional homogeneity rate (%)	
Binary interaction	2376	83.4	3127	74.2	$<10^{-4}$
3-motif	12087	66.4	39742	51.4	$<10^{-4}$
4-motif	98409	47.6	676682	32.1	$<10^{-4}$

[a] The “functional homogeneity rate” is calculated as the number of motifs whose members share at least one common functional category divided by the total number of motifs.

Table S39 Topology distribution of motifs whose members of the same age class and those of different age classes. The motif topologies are classified based on the topological saturation measured by the number of edges within the motif. The result is based on the PIN of DIP_YEAST_CORE. (A) 3-motif. (B) 4-motif. Rank sum test, $P<10^{-4}$ for both 3-motif and 4-motif.

	The number of motifs	
	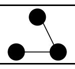	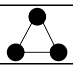
3-motifs whose members are of the same age class	11065	1022
The other 3-motifs	38192	1550

(A)

	The number of motifs			
	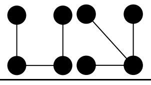	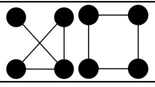	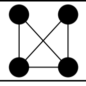	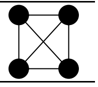
4-motifs whose members are of the same age class	81900	14015	1971	523
The other 4-motifs	627426	44825	3647	784

(B)

Table S40 The fraction of motifs within protein complexes among the motifs with constituents of the same age class and that among the motifs of different age classes. The result is based on the PIN of DIP_YEAST_CORE.

	Motifs whose members are of the same age class		Motifs whose members are of different age classes		*P*-value (Chi-squared test)
	Total number	The fraction of motifs within protein complexes (%)	Total number	The fraction of motifs within protein complexes (%)	
3-motif	12087	6.60	39742	3.32	$<10^{-4}$
4-motif	98409	1.80	676682	0.61	$<10^{-4}$

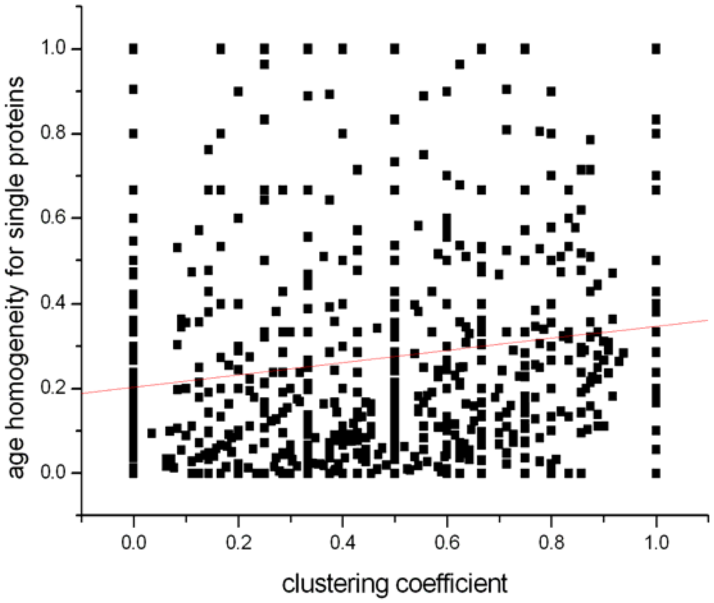

Figure S1 The scatter plot for clustering coefficient versus age homogeneity for single proteins. The age homogeneity for single proteins is defined as the fraction of its interaction partners which are of the same age class as the protein. It can weigh the degree of co-origins between the protein and its neighbors. The clustering coefficient of a node is computed as $C = \frac{2n}{k(k-1)}$, where n denotes the number of interactions between the k neighbors of the node. Pearson correlation analysis, r=0.14, $P<10^{-4}$.

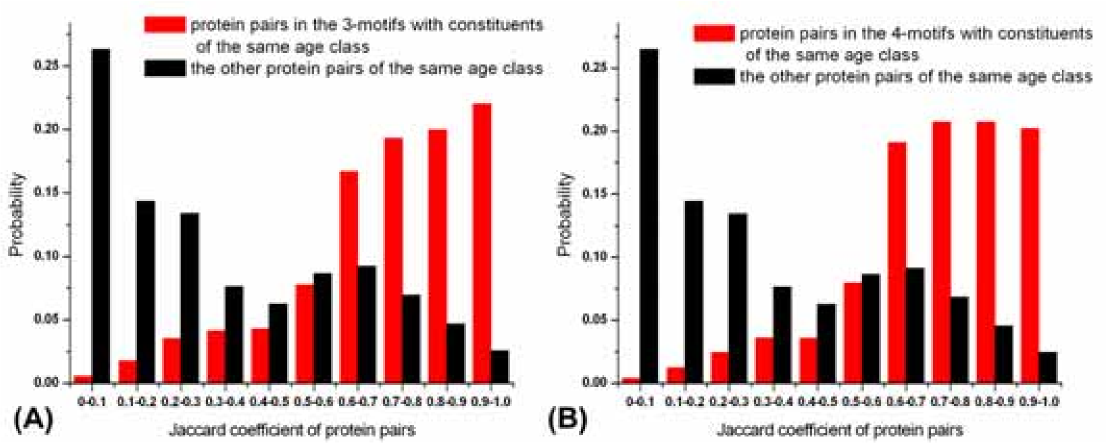

Figure S2 Distributions of Jaccard coefficients of protein pairs within motifs whose constituents are of the same age class and of the other protein pairs of the same age class. The Jaccard coefficient (*JC*) is used to measure the similarity between the phylogenetic profiles of protein pairs [6], which is computed as $JC_{ij} = \frac{n_{ij}}{n_i + n_j - n_{ij}}$, where n_i and n_j are the number of genomes that code the protein *i* and *j* respectively, and n_{ij} is the number of genomes that code both the two proteins. **(A)** 3-motif. Average *JC* is 7.2×10^{-1} for protein pairs in 3-motifs whose constituents are of the same age class, 3.5×10^{-1} for the other protein pairs of the same age class. Rank sum test, $P<10^{-4}$. **(B)** 4-motif. Average *JC* is 7.3×10^{-1} and 3.4×10^{-1} for the two classes of protein pairs respectively. Rank sum test, $P<10^{-4}$. Results are based on the PIN of DIP_YEAST_CORE.

References

1. Kunin V, Pereira-Leal JB, Ouzounis CA: **Functional evolution of the yeast protein interaction network.** *Mol Biol Evol* 2004, **21**: 1171-1176.
2. Albà MM, Castresana J: **Inverse relationship between evolutionary rate and age of mammalian genes.** *Mol Biol Evol* 2005, **22**: 598-606.
3. Wolf YI, Novichkov PS, Karev GP, Koonin EV, Lipman DJ: **Inaugural Article: The universal distribution of evolutionary rates of genes and distinct characteristics of eukaryotic genes of different apparent ages.** *Proc Natl Acad Sci U S A* 2009, **106:** 7273-7280.
4. Wheeler DL, Barrett T, Benson DA, Bryant SH, Canese K, Chetvernin V, Church DM, DiCuccio M, Edgar R, Federhen S, Geer LY, Helmberg W, Kapustin Y, Kenton DL, Khovayko O, Lipman DJ, Madden TL, Maglott DR, Ostell J, Pruitt KD, Schuler GD, Schriml LM, Sequeira E, Sherry ST, Sirotkin K, Souvorov A, Starchenko G, Suzek TO, Tatusov R, Tatusova TA, *et al.*: **Database resources of the National Center for Biotechnology Information.** *Nucleic Acids Res* 2006, **35**(Database issue): D5-12.
5. Mewes HW, Frishman D, Guldener U, Mannhaupt G, Mayer K, Mokrejs M, Morgenstern B, Munsterkotter M, Rudd S, Weil B: **MIPS: A database for genomes and protein sequences.** *Nucleic Acids Res* 2002, **30**: 31–34.
6. Zhao J, Ding GH, Tao L, Yu H, Yu ZH, Luo JH, Cao ZW, Li YX: **Modular co-evolution of metabolic networks.** *BMC Bioinformatics* 2007, **8**:311.
